# Effectiveness of exercise prehabilitation before anterior cruciate ligament reconstruction on functional outcomes – a single-blinded randomized controlled trial

**DOI:** 10.1038/s41598-026-41576-2

**Published:** 2026-03-09

**Authors:** Rebecca Abel, Daniel Niederer, Alexander Glowa, Niklas Hansen, Christiane Wilke, Christoph Offerhaus

**Affiliations:** 1 Institute of Movement Therapy and movement-oriented Prevention and Rehabilitation, Abt. 1., German Sport University Cologne, Am Sportpark Müngersdorf 6, 50933 Cologne, Germany; 2 Institute of Occupational, Social and Environmental Medicine, Goethe University Frankfurt, Theodor-Stern-Kai 7, 60590 Frankfurt, Germany; 3 PhysioSport PACE GmbH, Schanzenstraße 33, 51063 Cologne, Germany; 4 Department of Orthopedic Surgery and Sports Traumatology, Sana Medical Centre Cologne, Aachener Str. 445-449, 50933 Cologne, Germany; 5 Witten/Herdecke University, Alfred-Herrhausen-Str. 50, 58455 Witten, Germany

**Keywords:** knee, prehabilitation, preoperative training, ACL, RCT, intervention, self-administered, guided, training, Rehabilitation, Reconstruction

## Abstract

Objective: To compare the effectiveness of an individually tailored, adaptive, guided, structured and criteria-based preoperative rehabilitation program (IG) to a structured, non-guided, self-administered home training program (CG) in individuals after an anterior cruciate ligament (ACL) rupture. Methods: We enrolled participants 16 to 60 years with a unilateral ACL rupture, scheduled for reconstruction using a hamstring or quadriceps tendon autograft. They were assessor-blinded 1:1 block-randomized to the IG or the CG. Self-reported knee function, assessed using the Knee injury and Osteoarthritis Outcome Score (KOOS), was the primary outcome. Secondary outcomes included functional measures (range of motion, muscle strength, functional limb asymmetries, and hopping ability) as well as self-reported psychological readiness to return to sport. Parameters were assessed at up to seven time points: anamnesis in the hospital, 1 to 7 days before the surgery, on the day of surgery, 30, 60, 90, and 180 days post-surgery. Intention-to-treat based linear mixed models were calculated using the change scores since anamnesis. Results: Of 114 enrolled participants (mean age = 31.03, SD = 10.30 years; 53% females), 58 participants discontinued intervention or dropped out. The IG (KOOS score anamnesis: 46.04, 95% confidence interval = [45.07 to 47.02]; pre-reconstruction: 58.52, [57.51 to 59.54]) demonstrated a more pronounced preoperative improvement in the KOOS score compared to the CG (51.01, [50.10 to 51.92]; 59.18, [58.40 to 59.96]). The interaction (group*time) of the KOOS sum score differed for the change score to the surgery day (*p* = 0.039) and to 60 days postoperative (*p* = 0.039). Conclusion: The individually tailored preoperative rehabilitation program may improve perceived knee function more than self-guided training. Caution and future research are needed as the effects were small.

**Trial registration**: German Register of Clinical Trials, DRKSID: DRKS00030312, Date of registration in DRKS 26.09.2022.

**Supplementary Information:**

The online version contains supplementary material available at 10.1038/s41598-026-41576-2.

## Introduction

Athletes returning to sports (RTS) after an anterior cruciate ligament (ACL) rupture have up to a six-fold increased risk of experiencing a recurrent injury within two years postoperatively compared to uninjured athletes^1^. After RTS, such athletes often perform at a lower level and display shorter careers compared to their uninjured counterparts^2^. Secondary and follow-up complications often occur after surgical reconstruction, despite the implementation of adequate rehabilitation treatments^3^. As a result, the rehabilitation process following ACL ruptures requires enhancement to more effectively restore knee function and promote sustained physical activity.

After an ACL rupture, interventions performed until the reconstruction, so-called preoperative trainings or prehabilitation, aim to improve knee joint function including joint mobility and quadriceps strength, to enhance leg axis stability, to improve conditional and coordinative performance and perceived self-efficacy. This preoperative therapy approach may be linked to improvements in both pre- and postoperative functional outcomes, as well as return-to-sport rates, for up to two years after surgery^4–8^.

Despite these promising results on the effects of pre-operative interventions after an ACL rupture high-quality evidence regarding the effects of preoperative training is limited. Studies often offer only low to moderate quality evidence that prehabilitation approaches, particularly preoperative functional training, have a beneficial impact on both preoperative and postoperative functional performance^6^. Additionally, there is only low-quality evidence indicating that preoperative training positively affects quadriceps strength, performance in single-leg hop jumps three months postoperatively, self-reported knee function, and the speed of return to competition^6,9^. Based on the available evidence, an appropriate comparator group, sufficient follow-up duration, or essential outcome measures to provide robust evidence regarding the significance of preoperative rehabilitation interventions for addressing potential secondary issues would be needed to provide further robust evidence on the effectiveness of prehabilitative measures after ACL ruptures^6,9,10^. High-quality research is essential to substantiate this approach^7^. Furthermore, there is no evidence-based consensus on the optimal content, level of supervision such as one-on-one guidance versus self-administered training, and overall framework for preoperative training^6,8–11^.

The objective of our randomised controlled trial was to compare the effectiveness of a guided, structured and criteria-based preoperative rehabilitation program (adaptable to the individual’s performance level) to a non-guided and self-administered home-based training program on self-reported knee function (KOOS score) and additional functional outcomes (range of motion, muscle strength, functional limb asymmetries, and hopping ability) as well as self-reported psychological readiness to return to sport. We hypothesize that the guided prehabilitation program is more effective than self-administered home training in terms of its impact on the change score of the Knee Injury and Osteoarthritis Outcome Score (KOOS) from baseline to pre-reconstruction.

## Methods

### Design, randomisation and ethics

This study was performed as a monocentric, prospective randomized controlled single-blinded trial (Computer-generated block randomization, 1:1 allocation). To ensure random and blinded allocation, sealed envelopes containing the assignment information were opened by the allocator only after a participant had been enrolled in the study.

The study was approved by the ethics committee of the German Sport University on June 14, 2022 (ethics application no. 093/2022) and prospectively registered (DRKS-ID: DRKS00030312; date of registration: 26.09.2022). All experiments were performed in accordance with relevant guidelines and regulations.

The main outcome was the KOOS sum score. The study protocol with further details (for example secondary outcomes, sample size calculations) was published prospectively^12^(compare Supplement A.1). Minor changes to the study protocol are also provided in the Supplement (A.2).

Inclusion started in April 2023 and ended in June 2024, data collection ended in December 2024.

All participants provided written informed consent, but could withdraw their consent for study participation at any time without giving reasons and without affecting subsequent treatment.

### Participants

Potentially eligible individuals were recruited at Sana Medical Centre in Cologne. Participants were eligible if they were aged 16 to 60 years with a unilateral and complete primary ACL rupture (confirmed by magnetic resonance imaging and clinical examination) and indication for an arthroscopically assisted anatomic ACL reconstruction using a hamstring or quadriceps tendon autograft (inpatient or outpatient). The graft was selected using a standardised approach based on current recommendations^13^. Hamstring tendons were the primary graft choice. Whenever possible, a quadrupled semitendinosus (ST) graft was used. If the harvested ST tendon measured less than 28 cm in length, the gracilis tendon was additionally harvested to create either a quadrupled ST/gracilis (4×ST/G) or a six-strand ST/gracilis (6×ST/G) construct. Indications for the use of a quadriceps tendon graft, aside from concomitant medial collateral ligament (MCL) injuries, which constituted an exclusion criterion (see exclusion criteria), included valgus malalignment, participation in sports associated with high valgus loading of the knee (e.g., judo), or patient preference. Participants were included only if the duration between the inclusion and surgery was at least three weeks.

Participants with concomitant injuries with an intrinsic indication for surgery that lead to a change in the rehabilitation protocol and participants with prior operations or injuries to the knee joint (both to the injured leg and to the contralateral leg) in the last five years were excluded. Comorbidities that contraindicate participation in an active training program e.g., severe cardiovascular diseases or neurological disorders, along with underlying rheumatic diseases and pregnancy, were also considered as exclusion criteria.

If unscheduled procedures had to be performed during the surgical treatment that resulted in a modification of the postoperative therapy protocol (e.g., meniscus repair), the affected participants were considered dropouts from that point forward.

### Equity, diversity, and inclusion statement

A balanced and diverse recruitment of participants was ensured, as eligibility was not restricted by gender, ethnic/cultural background, or physical activity level. Women, as a marginalized group, were specifically included. The analysis accounted for both gender and activity level. Additionally, diversity was reflected within the research team, which included representatives of both genders, early-career researchers and senior professors, as well as experts from various disciplines (sports scientists, medical doctors, and therapists).

### Patient and public involvement

Participants and the public were not involved in the conception or the design of the study or the development of the research questions. During study conduction, all participants in the tailored intervention were actively included in the scheduling of the dose, exercise selection and progression. All home-base comparator participants selected the type (based on available exercises), dose, and volume of the exercises by their own.

### Interventions

After the ACL rupture, anamnesis/testing at the hospital and randomized allocation, participants performed the prehabilitation program as a part of the intervention group (guided prehabilitation program) or of the comparator group (self-administered home training). The training commenced at the earliest feasible date and extended through the reconstruction period, with a minimum duration of three weeks. The interval between rupture and surgery along with all medical decisions such as surgery timing and graft selection were determined as a part of the formal, medically prescribed reconstruction process and performed independently of the present study.

### Prehabilitation program (intervention group)

The participants of the intervention group performed a structured, criteria-based, guided training in a medical rehabilitation centre. The training program was tailored to the individual performance level and consisted of two 60-minute sessions per week. The intervention was supervised and individually performed. Additionally, participants were provided with exercises once per week for self-guided training at home. The intensity, volume, and (thus) type of the exercises in the prehabilitation program were increased progressively and includes the following components^6,14^:

- Mobilization of the knee joint, improvement of mobility.
- Improvement of neuromuscular control, especially of the musculus quadriceps.
- Improvement of postural stability and balance.
- Restoration or increase of muscle strength, especially knee flexors and extensors, abductors, plantar flexors; local muscle strength endurance training, increase of muscle section, increase of neuromuscular strength.

The selection of exercises aligns with those used in previous studies^6^. The detailed exercise selection and progression are shown in figure A.4 (Supplement). The prehabilitation program is criteria-based and includes exercises at two levels (Level I with basic exercises and Level II with progressively advanced exercises). At the outset, therapists at the rehabilitation facility tailored Level I exercises to the participants’ individual performance levels. If participants demonstrated full, active knee joint extension, experienced no tissue reaction the day following training for at least two consecutive sessions and reported no painful movement restrictions, the therapist would expand or replace exercises with those from Level II. Pain was assessed with a visual analog scale (VAS) of 0–10 for subjective pain assessment, with VAS < 3–4 as a framework for orientation within the training.

No time limit was imposed for reaching a specific level, and variations in participants’ performance were anticipated. Consequently, some participants might not complete all Level II exercises before surgical reconstruction and might only perform Level I exercises. The primary aim of the training program was to enhance individual performance rather than to attain a predefined performance level.

### Comparator: Self-administered home training

In clinical care participants typically do not receive supervised, criteria-based active therapy prior to surgical treatment; they are often instructed to exercise independently. Participants in the comparator group were similarly asked to engage in self-directed exercise and were provided with a brochure containing six exercises: active knee extension, single-leg stands, external hip abduction, hip bridge in a supine position, double-leg calf raises, and squats. These exercises aimed to achieve the same objectives as those in the guided prehabilitation program. The brochure included detailed instructions on exercise execution and recommended repetitions, as well as information on how to progress the exercises. Treating physicians at the hospital advised participants in the comparator group to perform these exercises three times a week as part of their self-administered preoperative home training. This self-directed training was chosen as the comparator to evaluate the effectiveness of different settings and levels of supervision on preoperative training following ACL injuries in this trial.

### Postoperative rehabilitation program

While the prehabilitation programs varied prior to surgery depending on the group, all participants performed the same standardized rehabilitation program following surgical reconstruction. Initially, quadriceps innervation and knee extension were practiced, along with patella mobilization. A pain-adapted, functional load progression was implemented. Functional exercises and strength training were gradually intensified and, from the 10th week onward, supplemented with jumping and rotational movements. The monitoring of rehabilitation concluded 180 days post-surgery.

### Outcomes

**Fig. 1:**
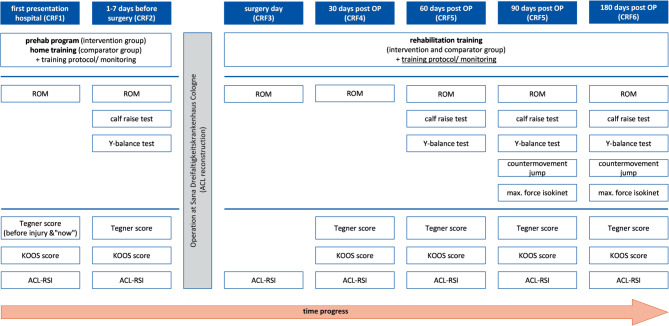
Participant timeline and testing-tools (CRF=case report file, OP =operation, ROM=range of motion, KOOS Score = Knee Injury and Osteoarthritis Outcome Score, ACL-RSI = anterior cruciate ligament - Return to Sport Injury Scale).

Functional parameters and self-reported knee function were assessed (not assessor-blinded) in both groups at seven specified measurement time points: during the initial hospital anamnesis, 1–7 days prior to ACL reconstruction, on the day of surgery, and at 30, 60, 90, and 180 days postoperatively (Fig. 1)^12^. Not all outcomes were assessed at each of the measurement days (Fig. 1).

The KOOS sum score serves as the primary outcome measure and assesses self-perceived knee function^15,16^. Furthermore, the following secondary outcomes were assessed: Range of Motion (RoM), particular the maximal RoM in extension and flexion was measured with a goniometer according to the neutral-zero method^17,18^. The strength of the knee flexors (hamstrings) and the knee extensors (musculus Quadriceps femoris) were recorded using an isokinetic device in a range of motion of 0° − 90° (maximum strength capacity = 5 repetitions at 60°/s, strength endurance = 15 repetitions at 180°/s) ^19^ (Biodex/System 4/Advantage BX Software 5.3.06). The strength of the plantar flexors (m. gastrocnemius) was tested with the “calf raise” test (single-leg stand and lift the heel off the ground maximally; number of repetitions (right and left) in full range of motion, with a maximum of 20 repetitions)^20^. Dynamic balance and the neuromuscular control and the strength of the lower extremity were measured through the Y-Balance Test. This test measures the reach of the playing leg in the three directions anterior, posteromedial and posterolateral (mobility of the joints: knee joints, hip joints, ankle joints)^21–23^. Jumping ability was assessed using the Counter Movement Jump in conjunction with a force plate (Contemplas/TwinPlate/Templo Version 2023.0.754). So the flight time and the impulse during the jump are determined^24^. Furthermore, the ACL-Return to Sport Injury Scale (ACL-RSI) was employed to assess self-confidence, risk perception and emotional responses related to the ACL injury alongside any complaints and issues arising from the knee joint injury^25^. Finally, participants’ activity levels were quantified using the Tegner score^26^. Further details on the measurements, outcomes, and conduction of the tests can be found in the published protocol^12^ and in the supplement (A.3).

### Statistical analysis

The analyses were performed on an intention to treat basis. To address missing data, multiple imputation was employed using IBM SPSS Statistics (Version 28). Dependent variables were ID, timepoint, group, age, gender, weight, Tegner Score pre-injury. This approach utilized chained equations in a fully conditional specification, executing 40 iterations to produce asymptotically unbiased estimations. For more Details see Supplement (A.6).

The two-sided alpha error threshold was set at 5% for all inferential statistical analyses, which were performed using R software (R version 4.3.3). Participants were categorized based on their group affiliation defined as a between-subject factor. The analyses were conducted using linear mixed models for repeated measurements. Inference statistical analyses were performed using the change scores of each outcome from baseline to each follow-up as the dependent variable, with these analyses being confirmatory in nature. Time effects, representing the repeated measures, were modelled as random effects, while other independent variables (group) and covariates (potential confounders and effect modifiers such as age, gender, and activity level) were treated as fixed effects. The same model was used to evaluate the influence of these clinically relevant confounders and effect modifiers on the treatment effect. For further details including the statistical code, see Supplement (A.7). The statistical analysis was designed and reported in accordance with the CHAMP statement^27^.

## Results

### sample

**Fig. 2:**
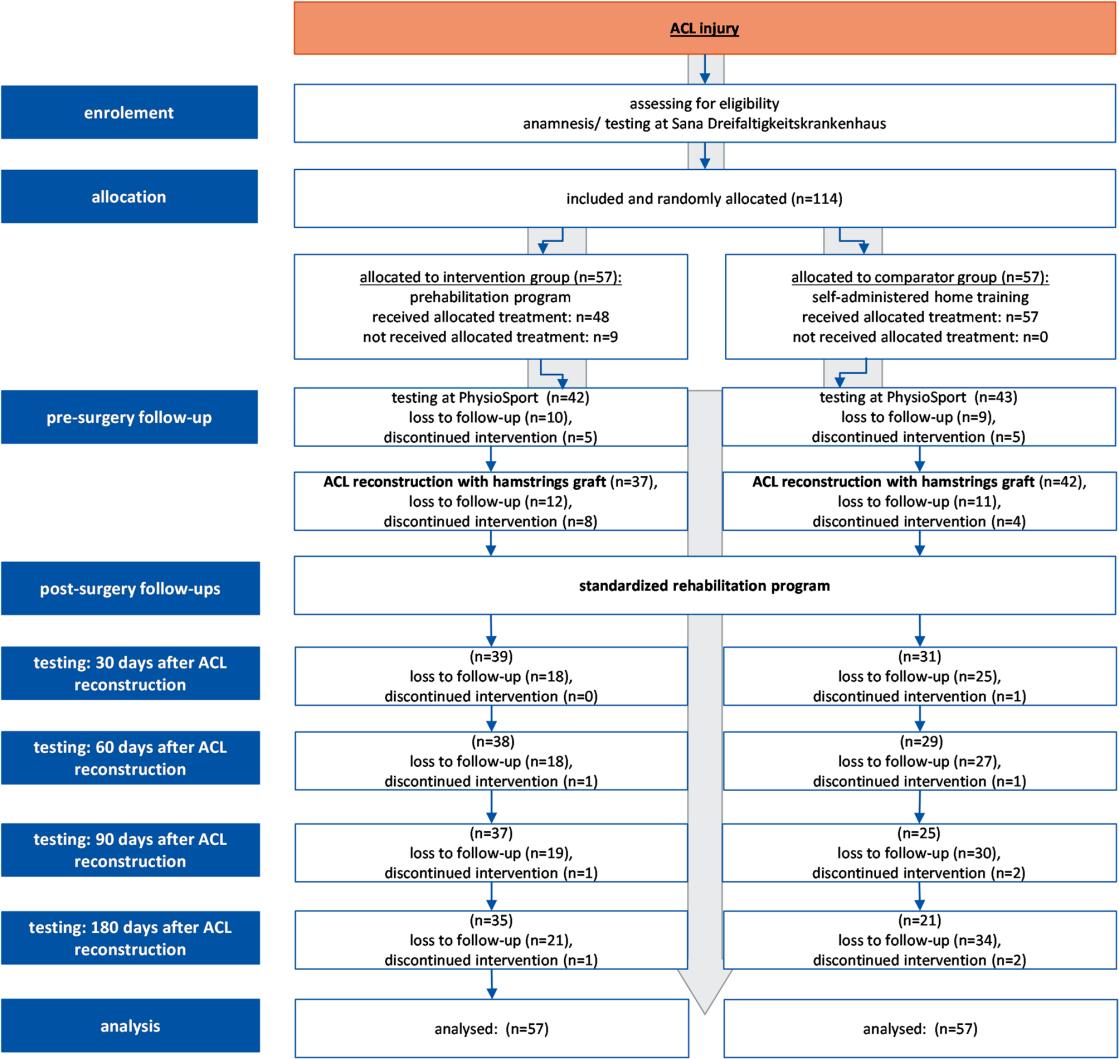
Study flow chart: study procedure (enrolement, allocation, pre-surgery follow-up, post-surgery follow-ups); (ACL = anterior cruciate ligament)

A total of 114 participants provided informed consent to participate in this study. The study’s progression including the number of participants randomized, those who received the intended treatment and those analysed for the primary outcome, is summarized in the flowchart (Fig. 2).

Of the 114 enrolled participants, 3 discontinued participation and 55 dropped out for the following reasons: lack of time (*n* = 15), difficulties accessing the rehabilitation facility due to distance (*n* = 6), familial reasons (*n* = 3), job reasons (*n* = 3), contact refused (*n* = 5), further knee operation (*n* = 1) and unspecified reasons (*n* = 18).

No serious adverse events were reported. However, adverse events necessitating exclusion were meniscus repair surgery (*n* = 7). Adverse events resulting in intervention interruption or missed visits included acute infections (*n* = 3), a fall on the stairs (*n* = 1) and pain-related inability to complete specific assessments (Y-Balance Test (*n* = 1), isokinetic testing (*n* = 7), Countermovement Jump (CMJ) testing (*n* = 8) and both CMJ and isokinetic testing (*n* = 7)).

### Baseline data

The sociodemographic profile, baseline and clinical characteristics of the study population in total and separated for each group are shown in Table 1; additional details can be found in the supplement (A.5).

**Table 1:** Sociodemographic profile, baseline and clinical characteristics of the participants; time of assessment of socio-demographic data: anamnesis in the hospital (t1); (n=number); Table 1a: M = mean, SD = standard deviation; Table 1b: percentage distribution; RoM = Range of Motion, QS = Quadriceps Tendon, ST = Semitendinosus Tendon, G = Gracilis Tendon, LET = Lateral Extra-articular Tenodesis.

Table 1a Domain	Outcome	Value/ unit	Total sample				Intervention group					Comparator group			
			n	Mean		SD	n	Mean		SD		n	Mean		SD
socio-demographic	age	years	114	31.03		10.30	57	31.18		11.07		57	30.88		9.56
	weight	kg	93	77.43		12.03	43	78.16		13.15		50	76.8		11.07
	height	cm	107	174.47		8.78	53	174.42		9.23		54	174.52		8.4
sports	training sessions before injury	number/ week	113	2.86		1.48	57	2.89		1.42		56	2.82		1.54
	training sessions before injury	minutes/ training	112	77.37		29.69	57	77.89		29.92		55	76.82		28.71
	Sport Level before injury	Tegner Score	88	6.51		1.58	46	6.43		1.52		42	6.6		1.65
	Sport Level	Tegner Score	82	2.61		1.44	39	2.31		1.59		43	2.88		1.26
RoM	extension deficit	degree (°)	108	5.45		6.62	54	6.28		5.9		54	4.63		7.22
	flexion difference, LSI	%	108	84.62		17.23	54	83.30		16.33		54	85.94		18.14
surgery	time between rupture & surgery	days	105	85.93		44.74	52	80.77		40.96		53	91.00		47.60
	days in the hospital	number	101	0.35		0.75	49	0.45		0.86		52	0.25		0.62

Table 1b Domain	Outcome	Value/ unit	Total sample				Intervention group				Comparator group				
			*n*		%		*n*		%		*n*			%	
socio-demographic	sex (*n* = 114)	male	60		52.63		29		25		31			27	
		female	54		47.37		28		25		26			23	
		non-binary	0		0		0		0		0			0	
sports	main sport (*n* = 111)	football	32		28.83		16		14.41		16			14.41	
		fitness	14		12.61		9		8.11		5			4.50	
		tennis	9		8.11		5		4.50		4			3.60	
		material arts	7		6.31		3		2.70		4			3.60	
		handball	5		4.50		2		1.80		3			2.70	
		dancing	7		6.31		3		2.70		4			3.60	
		others	37		33.33		18		16.22		19			17.12	
	Dominant leg (*n* = 106)	Left	20		18.87		13		12.26		7			6.60	
		right	86		81.13		41		38.68		45			42.45	
injury	injured limb (*n* = 114)	left	54		47,37		30		26.32		24			21.05	
		right	60		52,63		27		23.68		33			28.95	
	mechanism of injury (*n* = 114)	contact	18		15.79		11		9.65		7			6.14	
		non-contact	91		79.82		45		39.47		46			40.35	
		indirectly	5		4.39		1		0.88		4			3.51	
	sport of injury (*n* = 112)	football	37		33.04		18		16.07		19			16.96	
		tennis	6		5.36		3		2.68		3			2.68	
		skiing	26		23.21		13		11.61		13			11.61	
		material arts	5		4.46		2		1.79		3			2.68	
		dancing	5		4.46		2		1.79		3			2.68	
		accident (non-traffic)	6		5.36		4		3.57		2			1.79	
		others	27		24.11		13		11.61		14			12.50	
quadriceps activation	active stretch lifting (*n* = 106)	possible alone	105		99.06		54		50.94		51			48.11	
		possible with help	1		0.94		1		0.94		0			0	
		not possible	0		0		0		0		0			0	
surgery	meniscus repair (*n* = 101)	yes	7		6.93		2		1.98		5			4.95	
		no	94		93.07		47		46.53		47			46.53	
	graft (*n* = 101)	QS	6		5.94		4		3.96		2			1.98	
		4xST	54		53.47		24		23.76		30			29.70	
		4xST/G	28		27.72		16		15.84		12			11.88	
		6xST/G	2		1.98		0		0		2			1.98	
		QS + LET	1		0.99		1		0.99		0			0	
		ST + LET	7		7.93		3		2.97		4			3.96	
		ST/G + LET	3		2.97		1		0.99		2			1.98	

### Adherence to the protocol

Adherence to the protocol in the intervention and comparator groups was not different between groups; however, the variation in the intervention group (supervised sessions: M = 6.38, SD = 3.24, Min = 1, Max = 17) was higher than in the comparator group (self-administered sessions: M = 6.75, SD = 13.22, Min = 0, Max = 91).

### Imputation

The additive per-protocol analysis for the KOOS sum score is provided in the supplement (A.8).

For the KOSS score between 229 values (34.91%) and 244 values (37.20%) were imputed per item (S1–7, P1–9, A1–17, SP1–5, Q1–4) across all time points. Furthermore, multiple imputation was performed for Range of Motion, ACL-RSI, calf raise test and Y-Balance test LSI. For more details see Supplement (A. 6).

### Intervention effects

**Fig. 3:**
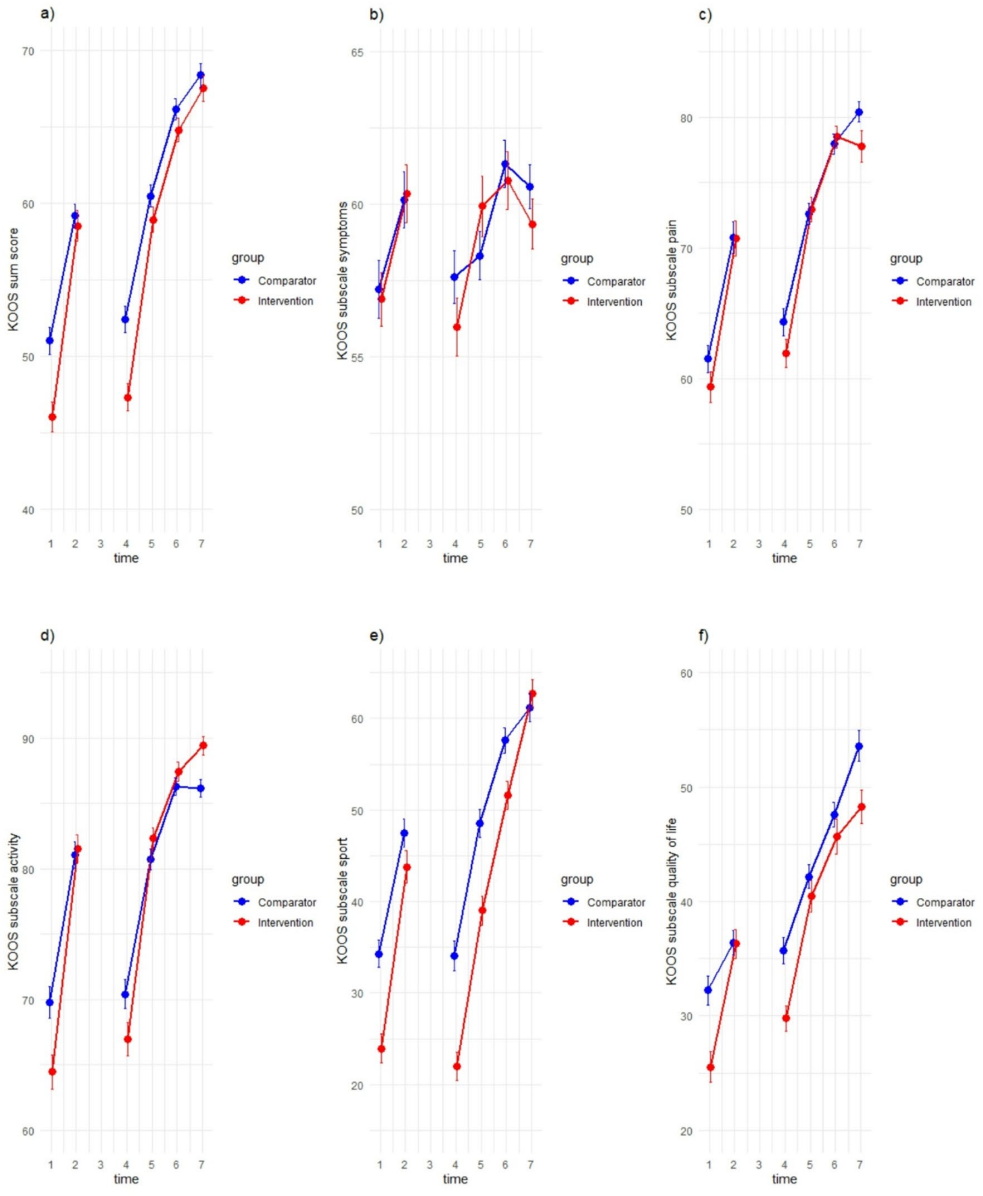
Means and 95% confidence intervals (CI) of the KOOS sum score and the KOOS subscales, separated by groups. The x-axes show the 7 measurement time points (t1 = during the initial hospital anamnesis, t2 = 1–7 days prior to ACL reconstruction, t3 = on the day of surgery, t4 = 30 days postoperatively, t5 = 60 days postoperatively, t6 = 90 days postoperatively and t7 = 180 days postoperatively), the y-axes show the values of the KOOS sum score (0-100%), the subscale (0-100%) and 95% CI; (KOOS = Knee Injury and Osteoarthritis Outcome Score); (a) KOOS sum score, (b) subscale symptoms, (c) subscale pain, (d) subscale activity, (e) subscale sport, (f) subscale quality of life.

**Fig. 4:**
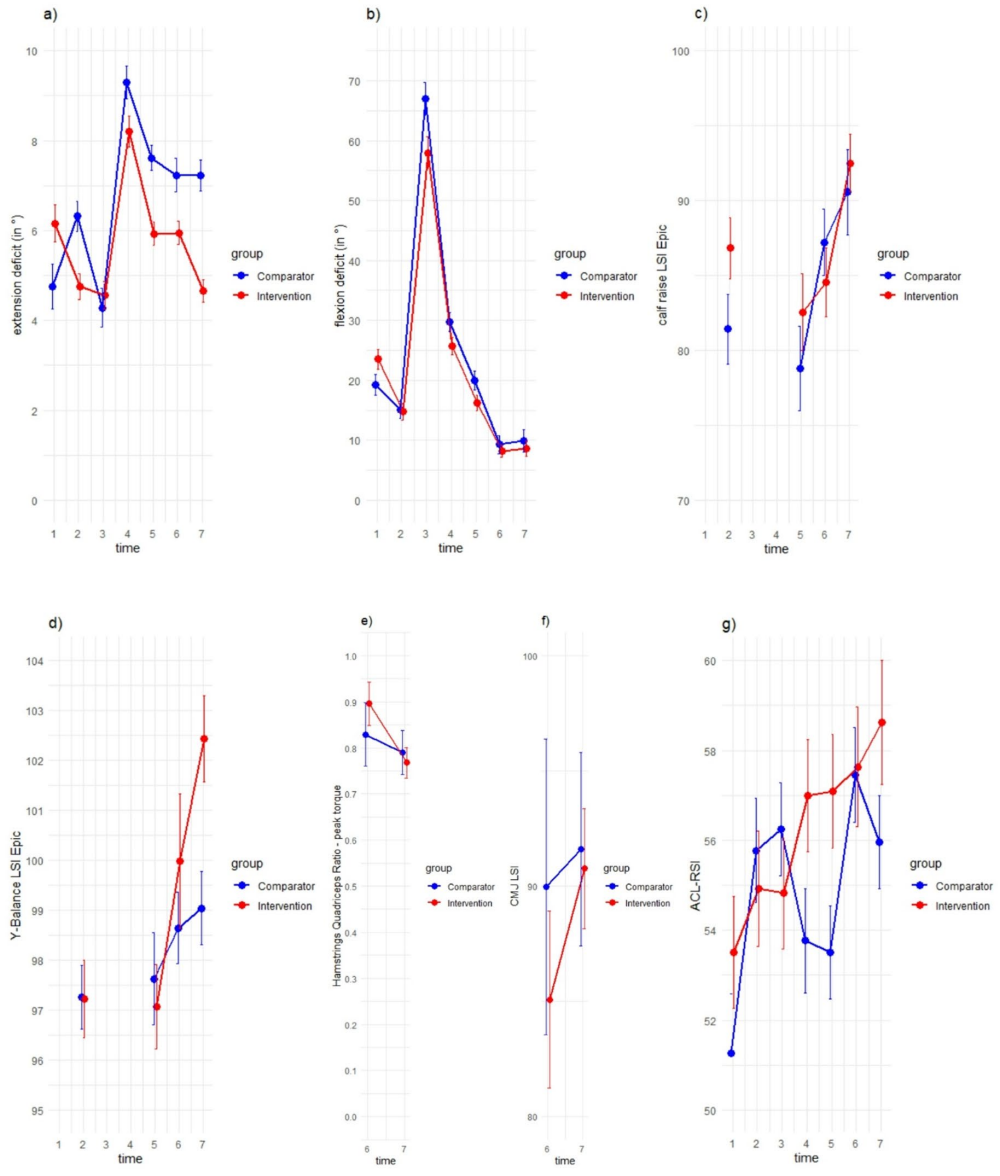
Means and 95% confidence intervals (CI) of the secondary outcomes, separated by groups. The x-axes show the 7 measurement time points (t1 = during the initial hospital anamnesis, t2 = 1–7 days prior to ACL reconstruction, t3 = on the day of surgery, t4 = 30 days postoperatively, t5 = 60 days postoperatively, t6 = 90 days postoperatively and t7 = 180 days postoperatively), the y-axes show the values and 95% CI; (a) extension deficit (in °), (b) flexion deficit (in °), (c) calf raise limb symmetry index with estimated pre-injury capacity (LSI Epic), (d) Y-Balance test (LSI Epic), (e) isokinetic: Hamstrings-Quadriceps-Ratio (HQ-Ratio) Peak Torque (60°), (f) Counter Movement jump (CMJ) (LSI), (g) Anterior Cruciate ligament Return to Sport Injury Scale (ACL-RSI).

The changes of the KOOS sum score and the KOOS subscales are illustrated in Fig. 3. Figure 4 shows the secondary outcomes (a) ACL-RSI, (b) extension deficit, (c) flexion deficit, (d) calf raise (limb symmetry index (LSI) with estimated pre-injury capacity (EPIC)), (e) Y-Balance (LSI EPIC), (f) isokinetic: Hamstrings-Quadriceps (HQ)-Ratio (Peak Torque), (g) CMJ jump (LSI).

Table 2 shows the output of the hypothesis answering primary mixed models for the main outcomes (KOOS sum score) from anamnesis to pre-surgery day. All main effects (time, group), interaction (time*group) effects and the contribution of the covariates are displayed. The groups differed at baseline (*p* < 0.001) and an interaction effect occurred (KOOS Baseline * time point pre-surgery) (*p* < 0.001). There are no significant effects for group, time and interaction (time*group), but the intervention group (mean KOOS sum score baseline: M = 46.04, CI 45.07–47.02; pre-surgery: M = 58.52, CI 57.51–59.54) shows a more pronounced improvement in the KOOS sum score than the comparator group (mean KOOS sum score baseline: M = 51.01, CI 50.10–51.92; pre-surgery: M = 59.18, CI 58.40–59.96).

**Table 2:** Results of linear mixed models, focusing on time points 1–2 for hypothesis testing.

Fixed effects	Estimate	Standard Error	Degrees of freedom	t-value	*P*-value
(Intercept)	-40.5	17.1	< 0.001	-2.37	1
group	< 0.001	6.213	220	0	1
time point	40.46	10.82	< 0.001	3.741	1
KOOS sum score Baseline	1	0.09	220	11.06	< 0.001***
group*KOOS sum score Baseline	-0	0.124	220	0	1
KOOS sum score Baseline*time point 2	-0.63	0.128	220	-4.95	< 0.001***
group*time point 2	8.903	8.787	220	1.013	0.312
Group*KOOS sum score Baseline*time point 2	-0.17	0.175	220	-0.96	0.337

* Indicate significant contributors; (KOOS= Knee Injury and Osteoarthritis Outcome Score)

All outcomes of the overall mixed model analyses for the KOOS sum score including main effects (time, group), interaction (group*KOOS-Baseline, group*time, group*KOOS-Baseline*time) as well as the contribution of the covariates for all time points are displayed in the Supplement (A.9). The interaction effect (group*time) of the KOOS sum score reached significance for the change score from time point 1 to time point 4 in favour of the CG (*p* = 0.039) and to time point 6 in favour of the IG (*p* = 0.039). The interaction effect (group*time) of the KOOS subscales is significant for the subscale symptoms for the change score from time points 1 to 6 in favour of the CG (*p* = 0.043), for the subscale activity for the change score from time point 1 to 4 in favour of the IG (*p* = 0.027) and for the subscale quality of life for the change score from time point 1 to 4 (*p* = 0.049), to 6 (*p* = 0.012) and to 7 (*p* = 0.043) all in favour of the IG. Further details are provided in the Supplement (A.10).

### Secondary intervention effects

The interaction effect (group*time) in the calf raise test was significant for the change score from time point 2 to 7 (*p* = 0.022) in favour of the CG (Supplement, A.14) and in the Y-Balance test for the change score from time point 2 to 6 in favour of the IG (*p* = 0.046) (A.15). There were no significant effects for the extension deficit (A.12), the flexion deficit (A.13), the ACL-RSI Scale (A.11), the Counter Movement Jump (A.17) and the isokinetic testing (A.16).

Additionally, the KOOS sum score (*p* = 0.009), the KOOS subscales pain (*p* = 0.007), activity (*p* < 0.001), sports (*p* = 0.001), the ACL-RSI (*p* = 0.005) and the height of CMJ (*p* < 0.001) were affected by the covariate age. The ACL-RSI (*p* = 0.002) and the height of CMJ (*p* < 0.001) were affected by gender. The calf raise test is affected by time point (*p* = 0.017). The HQ-ratio (isokinetic testing 60°) is affected by time between rupture and surgery (*p* = 0.013) and Tegner Score before injury (*p* = 0.022) (Supplement, A.9-A.17).

## Discussion

### Statement of principal findings

The conducted study highlights the effectiveness of a guided prehabilitation intervention prior to surgical ACL reconstruction in comparison to a self-administered home training program. Both, the guided training and the self-administered training group participants showed preoperative improvements in perceived knee function both prior to surgery and throughout the rehabilitative postoperative period. Although the intervention group began with considerably lower functional levels at baseline, it demonstrated more substantial improvement over time than the comparator group, up to the pre-reconstruction measurement. Similarly, the IG began at a lower level 30 days postoperatively, and tended to show larger gains throughout the subsequent follow-up period. These findings may indicate a potential benefit of guided, function-based training compared with self-administered home training, but the results should be interpreted with caution. The supervised, structured training is inevitably associated with increased contact with healthcare providers. The isolated effect of the training intervention is difficult to disentangle from the effect of augmented clinical contact. And it is important to consider that baseline differences between the groups (self-perceived knee function) meant the IG (with lower preoperative scores) had higher potential for improvement^28,29^.

### Sample representativeness

We included participants with an isolated, complete ACL rupture and an indication for ACL reconstruction using a hamstring or quadriceps tendon autograft. Prior to their injury the participants engaged in an average of almost three training sessions per week. This reflects a notably athletic cohort and the findings are generalizable to a young and physically active population.

### Adherence to the intervention

The total number of preoperative training sessions was not different in the two groups. However, the variation in the intervention group was much smaller than in the comparator group. All participants in the comparator group received the same recommendations from the treating therapists; however, some participants in the comparator group did not engage in independent training, other participants trained very frequently. Self-administered training offers greater flexibility and eliminates the need for travel to a training location. Additionally, athlete involvement and commitment in the planning, execution, and evaluation of training are important for the development of high training quality^30^. The key psychological factors associated with rehabilitation adherence include self-motivation, athletic identity and social support^31^. This was also shown in the present study: participants’ motivation and social support appear to be key factors influencing training frequency in the comparator group. Moreover, it is plausible that participants who struggled to motivate themselves for self-administered home training prior to surgery chose to withdraw from the study thereby introducing potential bias into the results.

In contrast, a strong therapeutic relationship is seen as a facilitator in rehabilitation^31^. Although guided training provides more time, as participants must travel to the training facility, the participants in the intervention group may have benefited from the therapeutic relationship. The therapist can monitor and correct the quality of exercise execution during guided training and thereby affecting the participants’ motivation positively. Particularly in the execution of challenging or uncomfortable exercises the therapist may have contributed to improving the quality in the intervention group, because an environment that promotes continuous and dynamic athlete-coach interaction is considered important for developing high training quality^30^. Additionally, the therapist can be an important support in load management^32^.

### Comparison with relevant literature

#### Perceived knee function of the participants

Compared to the percentile curves for the KOOS outcome score in a middle-aged Dutch population^33^ and compared to the KOOS values of a nationally record-based representative sample of 9996 adults derived from the Danish Civil Registration System^34^, the KOOS values of the participants in our study were quite low. For example, the values in the Danish study for the subscale Symptoms are M = 85 (95% CI: 85–86)^34^, whereas the mean scores in our study range from 56 to 61. This demonstrates the severity of ACL injuries, as impairments from the tear and the reconstruction of the ACL persist for an extended period (up to six months post-surgery).

#### Duration between injury and reconstruction

The duration between injury and surgical treatment is highly individual. The lower end of the preoperative period in this study, at three weeks, is relatively short. The organizational processes in the hospital necessitated this relatively short preoperative period in order to still achieve a sufficiently large sample size in this study. However, strength training can lead to increases in muscle strength after just a few weeks, which can in part be attributed to changes in the neural drive to the muscles, providing a rationale for the short duration^35^.

Overall, the time between injury and reconstruction averaged 86 days. However, the period in the comparator group was slightly longer than in the intervention group. Despite random allocation, differences between the intervention and comparator groups occurred. It is important to consider that numerous factors such as time between injury and reconstruction may have influenced the results^36^. Later-presenting non-acute anterior cruciate ligament-injured participants benefit more from surgical reconstruction^37^. The longer period in the comparator group may have improved the results in the comparator group. Thus, the more substantial improvement over a shorter period in the intervention group indicates a higher effectiveness of the guided training compared to self-administered training.

#### Psychological readiness

There were no differences between the groups in psychological readiness over time. The psychological readiness of the intervention group increased almost continuously, possibly due to the continuous feedback from the therapist, while the psychological readiness of the comparator group varied considerably. It is likely that psychological readiness depends on many different factors, so the intervention (guided vs. unguided preoperative training) had less influence on it. However, it was found that a higher age and male gender were associated with greater psychological readiness to return to sport. This is coherent to other studies that demonstrated that men show greater psychological readiness to return to sport than women^38^. The previous study results regarding age are heterogeneous. While in our study older age was associated with greater psychological readiness, other studies found that younger age was linked to higher psychological readiness^39^.

#### Knee extension/ flexion deficit

A postoperative knee extension deficit is not associated with age, sex, concomitant meniscus surgeries or other factors, but is primarily linked to preoperative extension and the time between injury and surgery^40^. Therefore, minimizing an extension deficit preoperatively is crucial for achieving good postoperative outcomes. While the extension deficit in the intervention group decreased, it increased in the comparator group. It is possible that the therapist was able to explicitly guide the participants during the supervised training to perform the exercises in high quality with a full range of motion, resulting in better outcomes in terms of range of motion^30^. Full knee extension after ACL injuries is often associated with pain, therefore, participants who performed self-administered training may not have completed the exercises with a full range of motion to achieve improvement.

#### Further functional and strength testing

No preoperative knee muscle strength measurements were obtained, even though strength is known to influence postoperative outcomes. Isokinetic strength testing is often not validly or feasibly assessable preoperatively in ACL-injured patients. Nevertheless, the functional tests that were conducted provide additional insights:

The LSI in the Y-Balance Test was consistently high across all measurement time points. With regard to the 90% threshold, all participants show over-threshold results^41^. Regarding the LSI in the counter movement jump (bilateral jump, ground reaction force in a side-to-side comparison) the 90% cut off value was also achieved for both groups 180 days post-op.

The gastrocnemius muscles support posture and forward progression during gait and their weakness can cause instability by impairing dorsiflexor counteraction^42^. The LSI calf raise test results indicate persistent strength asymmetries between the injured and uninjured leg throughout the observation period. Values exceed 90% in both groups only after 180 days, with the intervention group showing significantly higher values for the LSI than the comparator group. The improved preoperative values, likely resulting from guided training to enhance leg symmetry, may have an impact here, allowing for faster postoperative muscular symmetry.

A Hamstring-to-Quadriceps Ratio (HQ-Ratio) of 0.6 to 0.8 is generally considered healthy and functional^43^. Therefore, the measurements at time points 6 and 7 can be regarded as positive. Notably, the negative trend from time point 6 to 7 may be explained by an increase in quadriceps strength.

### Practical relevance

In clinical practice the time between injury and surgery is often spent passively. An active training program is strongly recommended. While the benefits of self-administered training include flexibility and autonomy in structuring the training, a guided training program may enhance the quality of exercise execution and accountability. Therefore, the decision to choose between guided or self-administered training should be based on the individual characteristics of the participants including their motivation and external circumstances.

### Methods discussion and limitations

#### KOOS Score

The population examined in our study is relatively young, with an average age of 31 years, and consists of physically active individuals. But the established five-factor structure of the KOOS does not fully apply to a sample of young, active participants undergoing ACL reconstruction, suggesting limited structural validity in certain subscales^44,45^. Factor analyses indicated that the five-factor structure did not fit adequately for this population, suggesting that a revised four-factor structure might be more suitable^44^. Moreover, calculating a sum KOOS score in the German version is not recommended^16,46^ and its weakness arises from its weak-to-moderate reliability^47^. Despite these limitations the strength of the KOOS score lies in its ability to capture significant effect sizes for tracking outcomes over time^47^ and it continues to be widely recognized and commonly used to assess participants with ACL injuries^44^.

#### Limb Symmetry Index

A decline in performance as assessed by limb symmetry indexes is often observed in the unaffected leg due to a lack of training. The LSI’s frequently overestimate knee function after ACLR^48^. Training the unaffected side, however, can have beneficial effects on both the affected and unaffected sides^49^. To ensure consistency in LSI calculation, the initially recorded values of the unaffected side were consistently used as a reference and compared to the values of the affected side at each respective time point^48^.

#### Drop Out

Despite serious efforts against, our study experienced a high dropout rate. Several participants withdrew from the study due to the unexpected burden of traveling to the training sessions and testing appointments in the rehabilitation facility. Additionally, the long duration of the study with the final testing occurring 180 days post-ACL reconstruction may have contributed to the increased dropout rates. Furthermore, a number of participants opted for conservative treatment instead of surgery after their inclusion in the study, which also impacted the dropout rate. In cases where participants decided against surgery post-inclusion, the final testing was conducted prior to the originally scheduled surgery date, adhering to the intention-to-treat principle.

#### Follow-up

The final assessment in this study was conducted 180 days post-surgery. At this point most participants had not yet returned to competitive sports. A longer follow-up period could potentially provide additional insights regarding those who still have not resumed athletic activities. While recent studies typically conduct follow-ups up to three months after surgery our study extended the follow-up duration to 180 days. The follow-up period is therefore relatively long^6^.

#### Prehabilitation as a multimodal program

Our study specifically focuses on the exercise and training component within the context of prehabilitation. Prehabilitation, in general is regarded as a comprehensive process that may involve multiple preoperative interventions^50^. It is therefore possible that a multimodal program (incorporating exercise therapy, psychological components, stress management, etc.) could further enhance postoperative outcomes.

## Conclusion

Due to the severity of ACL injuries and the concomitant low perceived knee function preoperative training presents substantial potential. Both guided and self-administered preoperative training have a positive impact on the knee function following ACL injuries. Self-administered training offers greater convenience and eliminates the need for travel to a training location. In contrast, a strong therapeutic relationship is a facilitator in rehabilitation considered important for developing high training motivation and quality. Given that both groups received similar postoperative rehabilitation and due to baseline differences, it is not appropriate to make strong causal claims that the preoperative, guided intervention led to superior postoperative outcomes. The decision to choose between guided or self-administered training should be based on the individual characteristics of the participants including their motivation and external circumstances. Further combinations of supervised and self-directed training and enhancements to the training through a multimodal program, e.g. psychological components, are also conceivable. Future studies are warranted to determine dose-response relationships as well as the long-term and re-injury effects of the interventions.

## Supplementary Information

Below is the link to the electronic supplementary material.

Supplementary Material 1

## Abbreviations

ACL: anterior cruciate ligament
KOOS: Knee Injury and Osteoarthritis Outcome Score
ACL-RSI: anterior cruciate ligament - Return to Sport Injury Scale
VAS: visual analog scale
RoM: range of motion
rep.: repetitions
CRF: case report file
OP: operation
CMJ: Counter Movement Jump
QS: Quadriceps Tendon
ST: Semitendinosus Tendon
G: Gracilis Tendon
LET: Lateral Extra-articular Tenodesis
LSI: limb symmetry index
EPIC: estimated pre-injury capacity
HQ Ratio: Hamstrings-Quadricpes Ratio

## Declarations

### Data sharing

The data sets analysed in this study are chaired open access in a publicly available data repository (Research Gate, https://www.researchgate.net/publication/390201342_Data_file_-_Prehabilitation_before_anterior_cruciate_ligament_reconstruction).
