# Supplementary material for: Effectiveness of exercise prehabilitation before anterior cruciate ligament reconstruction on functional outcomes – a single-blinded randomized controlled trial
Source: Sci Rep. 2026 Mar 9;16:8962. doi: 10.1038/s41598-026-41576-2 (PMC12987922; doi:10.1038/s41598-026-41576-2)
Supplement: Supplementary file 1 — Supplementary Material 1 [file 41598_2026_41576_MOESM1_ESM.docx]

SUPPLEMENT

Effects of exercise prehabilitation before anterior cruciate ligament reconstruction on functional outcomes during pre- and postoperative rehabilitation – a single-blinded randomized controlled trial

Table of Contents

[1. A.1 sample size determination 3](#_Toc193296206)

[2. A.2 changes to trial protocol 3](#_Toc193296207)

[3. A.3 Test implementation 4](#_Toc193296208)

[4. A.4 Intervention details 7](#_Toc193296209)

[5. A.5 Participants characteristics 7](#_Toc193296210)

[6. A.6 Multiple Imputation 9](#_Toc193296211)

[7. A.7 Statistical Analysis Code 12](#_Toc193296212)

[8. A.8 Per-Protocol Analysis 14](#_Toc193296213)

[9. A.9 Mixed Models outcomes – KOOS sum Score 16](#_Toc193296214)

[10. A. 10. Mixed models outcomes – KOOS subscales 18](#_Toc193296215)

[10.1 Subscale Symptoms 18](#_Toc193296216)

[10.2 Subscale Pain 19](#_Toc193296217)

[10.3 Subscale activity 21](#_Toc193296218)

[10.4 Subscale sports 22](#_Toc193296219)

[10.5 Subscale quality of life 24](#_Toc193296220)

[11. A.11 Mixed Models Outcomes – ACL-RSI 25](#_Toc193296221)

[12. A.12 Mixed models outcomes – extension deficit 27](#_Toc193296222)

[13. A.13 Mixed models outcomes – flexion deficit 28](#_Toc193296223)

[14. A.14 Mixed models outcomes – Calf raise test 30](#_Toc193296224)

[14.1 Calf raise test (LSI Epic) 30](#_Toc193296225)

[14.2 Calf raise test (LSI) 31](#_Toc193296226)

[15. A.15 Mixed models outcomes – Y-Balance Test 33](#_Toc193296227)

[15.1 Y-Balance LSI Epic 33](#_Toc193296228)

[15.2 Y-Balance LSI 35](#_Toc193296229)

[16. A.16 Mixed models outcomes – isokinetic testing 37](#_Toc193296230)

[16.1 HQ-Ratio – Peak Torque (60°) 37](#_Toc193296231)

[16.2 HQ-Ratio – sum of torques (180°) 38](#_Toc193296232)

[17. A.17 Mixed models outcomes – CMJ 39](#_Toc193296233)

[17.1 CMJ- height 39](#_Toc193296234)

[17.2 CMJ – LSI 41](#_Toc193296235)

[18. A.18 Consort Checklist 43](#_Toc193296236)

[19. A.19 Checklist TIDier (Template for Intervention Description and Replication) 46](#_Toc193296237)

[20. A.20 Supplemental references 48](#_Toc193296238)

# A.1 sample size determination

“The sample size calculation is based on the sum score of the KOOS (primary outcome parameter). The difference in the KOOS score of the comparator and intervention group at the measurement pre-reconstruction will be compared. The minimal clinically important change (MIC) for the KOOS score is reported to be a change of 8–10 with respect to the subscale score in patients with knee injuries [1]. For performing a sample size calculation, usually, a standard deviation of SD = 15 is used for the KOOS score. On this basis, an effect size of d = 0.533 was calculated using G*Power [2]. With a power of 80% and a one-sided alpha error level of 2.5%, data must be collected from n = 114 participants (equal number of participants in comparator and intervention group of n = 57 participants).” [3]

# A.2 changes to trial protocol

According to the study protocol, all analyses were to be conducted exclusively using R. However, data imputation was performed using SPSS.

According to the study protocol, participants were included only if the duration between the injury and surgery was at least 3 weeks and no longer than 13 weeks. Due to scheduling constraints and participants preferences the interval between injury and surgery was extended beyond 13 weeks in some cases. This reflects the typical clinical practice for anterior cruciate ligament (ACL) injuries. As a result, participants were included if the time between injury and surgery ranged from 3 to 33 weeks.

According to the study protocol, after surgical treatment participants of both groups participated in a standard, guided, postoperative rehabilitation programme at PhysioSport. Due to limited access to rehabilitation facility for some participants following the surgical reconstruction (e.g. inability to drive, difficulty using public transportation) several participants from both groups completed the postoperative standardized rehabilitation program at alternative physiotherapy clinics. However, the content of the program (compare study protocol) remained consistent. The postoperative assessments (at 30, 60, 90, and 180 days post-surgery) were conducted as planned, all at the selected rehabilitation facility.

Workability was intended to be assessed by determining the number of days absent from work both preoperatively and postoperatively. However, the responses provided by the participants were occasionally inconsistent and it was not considered that many participants were students or were not employed at the time of the study. Consequently, workability was not evaluated as part of the study.

The activation of the quadriceps muscle was evaluated using the active stretch lifting test (= active lifting of the extended leg, evaluation: "not possible", "possible with help" or "possible alone"). Almost all participants completed the test independently at all time points making it of little additional value. Consequently, it was not included in the study analysis.

# A.3 Test implementation

The testing procedures are specified in detail in the study protocol as follows [3]:

“Functional parameters as well as self-reported knee function will be collected in both groups at seven defined measurement time points (anamnesis in the hospital, 1–7 days before ACL reconstruction, on the day of surgery, and 30, 60, 90, and 180 days postoperative). The primary time point is 1–7 days before ACL reconstruction.

*Primary outcome: self‑reported knee function*

The primary outcome of the study is the participants’ self-perceived knee function, which is measured by the Knee Injury and Osteoarthritis Outcome Score (KOOS). The KOOS score is a measurement tool to assess participants’ self-perceived knee function, especially after knee injuries such as ACL injuries [4, 5]. The KOOS score consists of 42 items in five subscales: pain, symptoms, function in daily living (ADL), function in sport and recreation (Sport/Rec) and knee-related quality of life (QOL). The mean of the subscale scores can be calculated and used to make an overall assessment of knee function [6]. The KOOS score has a high test– retest reliability. Overall, the minimum detectable change in patients with knee injuries is reported as 8–10. Since the KOOS score is used as the primary outcome in numerous randomised-controlled trials, there is a corresponding comparability of the results with other studies [5, 7]. Reference values of the KOOS score of patients after ACL reconstruction, of healthy subjects and active soccer players are also available and can be used for comparisons [8–11].

*Secondary outcomes*

*Functional outcomes*

*Range of motion*

The active mobility of the knee joint (max. extension and flexion) is measured with a goniometer according to the neutral-zero method both preoperatively (following the anamnesis and 1–7 days before surgery) and postoperatively (on the day of surgery, 30 days and 60 days postoperative). The mobility of the knee joint is represented in a three-part code indicating the max. extension, the zero position as well as the max. flexion (e.g. 5°/0°/110°).

*Neuromuscular control/activation*

During the anamnesis, 1–7 days before the surgical reconstruction, on the day of the surgical reconstruction and 30 days after the surgical reconstruction, the participants are asked to perform an active extension of the leg while sitting (long sitting), i.e. to lift the extended leg from a mat (active stretch lifting = active lifting of the extended leg). The physician or therapist performing the test evaluates the performance as “not possible”, “possible with help” or “possible alone”.

*Strength/torque (knee flexors and extensors, plantar flexors)*

The strength of the knee flexors (hamstrings) and the knee extensors (m. quadriceps) is recorded 90 and 180 days postoperative using a strength test on the isokinet in a range of motion of 0°–90° (RoM 0°/0°/90°). For this purpose, maximum strength capacity is tested with 5 repetitions at 60°/s and strength endurance with 15 repetitions at 180°/s.

The strength of the plantar flexors (m. gastrocnemius) is tested with the “calf raise test”. The participants stand uprightwith their legs extended and lift their heels off the floor to the maximum. Then one foot is lifted off the ground (single-leg stand) and the participants lift the heel off the ground maximally (plantar flexion). This test is performed both preoperatively (1–7 days before surgery) and postoperatively (after 60, 90 and 180 days). After 20 repetitions in full range of motion, the test is terminated. The therapist notes the number of repetitions the participant has completed in a side-by-side comparison (right/left) if the required number of 20 repetitions has not been reached.

*Dynamic postural control*

The dynamic postural control is measured by the Y-balance test. The Y-balance test is a functional and unilateral dynamic balance test in a single-leg stance and is performed by participants 1–7 days before surgery and 60, 90 and 180 days postoperative. It checks the range of motion of the playing leg in the three directions anterior, posteromedial and posterolateral (mobility of the joints: knee joints, hip joints, ankle joints) [12], the strength of the lower extremity [13], the proprioception [13], the dynamic balance [28] and the neuromuscular control, postural control, postural stability, as well as the trunk stability [13, 14]. The best values from both sides (left/right) and each direction (anterior/ posteromedial/posterolateral) are selected from the valid trials and a total score is calculated. If the total score for one of the legs or for both legs is lower than 94%, there is an increased risk of injury [14]. The total scores (left/ right) are used for further calculation of the LSI. The LSI (limb symmetry index) is used to determine whether a deficient lateral deviation is present [15]. Jumping ability Counter Movement Jump is used to assess jumping abilities in a slow lengthening-shortening cycle. It is performed at 90 and 180 days postoperative and tests the explosive power, the maximum strength and the vertical bounce [16]. With the aid of a force plate, the flight time and the impulse during the jump are determined so that the jump height can be calculated. In addition, the force development is recorded in relation to the body weight as well as the percentage difference of the maximum force development in the bilateral comparison of the legs (affected vs. unaffected leg). […]

*Return to sport - Psychological readiness*

The ACL-Return to Sport Injury Scale (ACL-RSI) is used to assess self-confidence, risk assessment, and emotions related to the ACL injury as well as complaints and problems caused by the knee joint injury. Participants answer a total of 12 questions on the 10-point scale from 0 to 100 1–7 days before surgery and 30, 60, 90, and 180 days postoperative [17]. A total score is determined from the results of the 12 questions answered (score achieved/number of questions). If the total score is below 51%, this indicates a lack of confidence in the knee joint. The decision to return to sport after an ACL injury is significantly related to response on the ACL-RSI scale [17].

*Return to sport — success*

The RTS success is characterised by achieving the pre-injury level of sports participation as defined by the same type, frequency, intensity, and quality of performance as before the injury. The participant’s activity level is assessed at an initial presentation at the hospital (anamnesis), 1–7 days prior to surgery and 30, 60, 90 and 180 days postoperatively using the Tegner score (11-point scale). The Tegner score was developed specifically to assess the activity level of participants with ACL injuries and rates it on a scale from 1 (low activity level) to 10 (professional level) [18–20].”

# A.4 Intervention details

Figure A.4 Exercise selection of the prehabilitation program (level I and level II), recommended number of repetitions and adaptation possibilities of the exercises to lower/higher performance levels. ROM = range of motion, VAS = visual analog scale, rep. = repetitions [3].

# A.5 Participants characteristics

*Table A.5.1 Additional details about table 1 (Sociodemographic profile, baseline and clinical characteristics of the participants): main sport before injury (percentage distribution; n = number; time of assessment: anamnesis in the hospital (t1))*

| **main sport** | **Total sample** | **Intervention group** | **Comparator group** |
| --- | --- | --- | --- |
| number of participants  football  fitness  running  tennis  gymnasics  skiing  material arts  handball  basketball  lacrosse  volleyball  pilates  bicycling  dancing  climbing  horse riding  swimming  hiking  acrobatics  rugby  badminton  yoga  cheerleading  hockey  others | n= 111  n=32 (28.83%)  n=14 (12.61%)  n=4 (3.60%)  n=9 (8.11%)  n=1 (0.90%)  n=4 (3.60%)  n=7 (6.31%)  n=5 (4.50%)  n=3 (2.70%)  n=1 (0.90%)  n=2 (1.80%)  n= 1(0.90%)  n=2 (1.80%)  n=7 (6.31%)  n=2 (1.80%)  n=1 (0.90%)  n=2 (1.80%)  n=2 (1.80%)  n=1 (0.90%)  n=1 (0.90%)  n=1 (0.90%)  n=1 (0.90%)  n=1 (0.90%)  n=1 (0.90%)  n=4 (3.60%) | n= 56  n=16 (14.41%)  n=9 (8.11%)  n=2 (1.80%)  n=5 (4.50%)  n=1 (0.90%)  n=2 (1.80%)  n=3 (2.70%)  n=2 (1.80%)  n=2 (1.80%)  n=1 (0.90%)  n=0 (0%)  n=0 (0%)  n=1 (0.90%)  n=3 (2.70%)  n=0 (0%)  n=1 (0.90%)  n=2 (1.80%)  n=0 (0%)  n=1 (0.90%)  n=1 (0.90%)  n=0 (0%)  n=1 (0.90%)  n=1 (0.90%)  n=0 (0%)  n=1 (0.90%) | n= 55  n=16 (14.41%)  n=5 (4.50%)  n=2 (1.80%)  n=4 (3.60%)  n=0 (0%)  n=2 (1.80%)  n=4 (3.60%)  n=3 (2.70%)  n=1 (0.90%)  n=0 (0%)  n=2 (1.80%)  n=1 (0.90%)  n=1 (0.90%)  n=4 (3.60%)  n=2 (1.80%)  n=0 (0%)  n=0 (0%)  n=2 (1.80%)  n=0 (0%)  n=0 (0%)  n=1 (0.90%)  n=0 (0%)  n=0 (0%)  n=1 (0.90%)  n=3 (2.70%) |

*Table A.5.2 Additional details about table 1 (Sociodemographic profile, baseline and clinical characteristics of the participants): sport of injury (percentage distribution; n = number; time of assessment: anamnesis in the hospital (t1))*

| **sport of injury** | **Total sample** | **Intervention group** | **Comparator group** |
| --- | --- | --- | --- |
| number of participants  football  fitness  running  tennis  gymnastics  skiing  material arts  handball  basketball  lacrosse  volleyball  traffic accident  bicycle  hiking  dancing  horse riding  sledding  accident (non-traffic)  trampoline  rugby  badminton  others | n=112  n=37 (33.04%)  n=0 (0%)  n=0 (0%)  n=6 (5.36%)  n=1 (0.89%)  n=26 (23.21%)  n=5 (4.46%)  n=4 (3.57%)  n=3 (2.68%)  n=1 (0.89%)  n=2 (1.79%)  n=1 (0.89%)  n=2 (1.79%)  n=3 (2.68%)  n=5 (4.46%)  n=1 (0.89%)  n=1 (0.89%)  n=6 (5.36%)  n=2 (1.79%)  n=1 (0.89%)  n=1 (0.89%)  n=4 (3.57%) | n=55  n=18 (16.07%)  n=0 (0%)  n=0 (0%)  n=3 (2.68%)  n=1 (0.89%)  n=13 (11.61%)  n=2 (1.79%)  n=1 (0.89%)  n=2 (1.79%)  n=1 (0.89%)  n=0 (0%)  n=1 (0.89%)  n=0 (0%)  n=2 (1.79%)  n=2 (1.79%)  n=1 (0.89%)  n=1 (0.89%)  n=4 (3.57%)  n=0 (0%)  n=1 (0.89%)  n=0 (0%)  n=2 (1.79%) | n=57  n=19 (16.96%)  n=0 (0%)  n=0 (0%)  n=3 (2.68%)  n=0 (0%)  n=13 (11.61%)  n=3 (2.68%)  n=3 (2.68%)  n=1 (0.89%)  n=0 (0%)  n=2 (1.79%)  n=0 (0%)  n=2 (1.79%)  n=1 (0.89%)  n=3 (2.68%)  n=0 (0%)  n=0 (0%)  n=2 (1.79%)  n=2 (1.79%)  n=0 (0%)  n=1 (0.89%)  n=2 (1.79%) |

# A.6 Multiple Imputation

**Imputation Specifications**

- At measurement time points 1 and 2, 1260 values (26.32%) were imputed for the KOOS score. At measurement time point 4, 1512 values (31.57%) were imputed, at time point 5, 1596 values (33.33%), at time point 6, 1806 values (37.72%), and at time point 7, 2184 values (45.61%).
- Imputation Method: Fully Conditional Specification
- Number of Imputations: 5
- Iterations of the fully Conditional Specification Method: 40
- Model for Metric Variables: Linear Regression
- Dependent variables: ID, timepoint, group, age, gender, weight, Tegner Score pre-injury
- Imputed variables and predefined ranges:
  - Range of Motion
    non-injured leg:
    - Extension: Min = 0, Max =20
    - Neutral: Min = 0, Max = 5
    - Flexion: Min = 0, Max = 180

Injured leg:

- - - Extension: Min =0, May =25
    - Neutral: Min = 0, Max = 40
    - Flexion: Min = 0, Max = 180
  - KOOS
    - Symptoms: S1-S7, pain: P1 – P9, activity: A1 – A17, sport: SP1 – SP5, quality of life: Q1-Q5
    - Min = 0, Max = 4; rounded numbers
  - ACL-RSI
    - ACL-RSI 1 – 12
    - Min = 0, Max = 10; rounded numbers
  - Calf raise test
    - Non-injured and injured leg
    - Min = 0, Max = 20; rounded numbers
  - Y-Balance, LSI (EPIC)
    - Min = 0, Max = 120

*Tab. A.6 Missing values and Imputed values (RoM = range of motion, KOOS = Knee injury and Osteoarthritis Outcome Score, ACL-RSI = anterior cruciate ligament - Return to Sport Injury Scale, LSI = limb symmetry index)*

|  | **Missing values** | **Imputed values in 5 imputations** |
| --- | --- | --- |
| RoM, non-injured leg, Extension | 249 | 1245 |
| RoM, non-injured leg, Neutral Position | 249 | 1245 |
| RoM, non-injured leg, Flexion | 249 | 1245 |
| RoM, injured leg, Extension | 249 | 1245 |
| RoM, injured leg, Neutral Position | 248 | 1240 |
| RoM, injured leg, Flexion | 248 | 1240 |
| KOOS Symptoms 1 | 345 | 1725 |
| KOOS Symptoms 2 | 345 | 1725 |
| KOOS Symptoms 3 | 346 | 1730 |
| KOOS Symptoms 4 | 346 | 1730 |
| KOOS Symptoms 5 | 346 | 1730 |
| KOOS Symptoms 6 | 345 | 1725 |
| KOOS Symptoms 7 | 345 | 1725 |
| KOOS Pain 1 | 346 | 1730 |
| KOOS Pain 2 | 348 | 1740 |
| KOOS Pain 3 | 346 | 1730 |
| KOOS Pain 4 | 346 | 1730 |
| KOOS Pain 5 | 347 | 1735 |
| KOOS Pain 6 | 346 | 1730 |
| KOOS Pain 7 | 345 | 1725 |
| KOOS Pain 8 | 346 | 1730 |
| KOOS Pain 9 | 345 | 1725 |
| KOOS Activity 1 | 346 | 1730 |
| KOOS Activity 2 | 345 | 1725 |
| KOOS Activity 3 | 345 | 1725 |
| KOOS Activity 4 | 345 | 1725 |
| KOOS Activity 5 | 346 | 1730 |
| KOOS Activity 6 | 346 | 1730 |
| KOOS Activity 7 | 349 | 1745 |
| KOOS Activity 8 | 349 | 1745 |
| KOOS Activity 9 | 346 | 1730 |
| KOOS Activity 10 | 347 | 1735 |
| KOOS Activity 11 | 346 | 1730 |
| KOOS Activity 12 | 347 | 1735 |
| KOOS Activity 13 | 347 | 1735 |
| KOOS Activity 14 | 346 | 1730 |
| KOOS Activity 15 | 346 | 1730 |
| KOOS Activity 16 | 351 | 1755 |
| KOOS Activity 17 | 349 | 1745 |
| KOOS Sport 1 | 350 | 1750 |
| KOOS Sport 2 | 357 | 1785 |
| KOOS Sport 3 | 360 | 1800 |
| KOOS Sport 4 | 351 | 1755 |
| KOOS Sport 5 | 348 | 1740 |
| KOOS Quality of life 1 | 347 | 1735 |
| KOOS Quality of life 2 | 346 | 1730 |
| KOOS Quality of life 3 | 346 | 1730 |
| KOOS Quality of life 4 | 347 | 1735 |
| ACL_RSI1 | 277 | 1385 |
| ACL_RSI2 | 275 | 1375 |
| ACL_RSI3 | 275 | 1375 |
| ACL_RSI4 | 274 | 1370 |
| ACL_RSI5 | 275 | 1375 |
| ACL RSI6 | 275 | 1375 |
| ACL_RSI7 | 275 | 1375 |
| ACL_RSI8 | 275 | 1375 |
| ACL_RSI9 | 275 | 1375 |
| ACL_RSI10 | 276 | 1380 |
| ACL_RSI11 | 275 | 1375 |
| ACL RSI12 | 275 | 1375 |
| calf raise test, injured leg | 503 | 2515 |
| calf raise test, non-injured leg | 503 | 2515 |
| Y Balance Test, LSI | 504 | 2520 |

# A.7 Statistical Analysis Code

library(openxlsx)

library(readxl)

library(lme4)

library(ggplot2)

library(dplyr)

library(lmerTest)

library(robustlmm)

(Exemplary for the main outcome)

data_real_filter <- data_real |>

filter(KOOS_Baseline != 0) |>

filter(!is.na(KOOS_gesamt))

real_KOOS_all_diff <- lmer(KOOS_Diff ~ Gruppe + Zeitpunkt + (1|Zeitpunkt) + KOOS_Baseline*Gruppe*as.factor(Zeitpunkt), data = data_real_filter)

real_KOOS_Kovariate_diff <- lmer(KOOS_Diff ~ KOOS_Baseline + Gruppe + Zeitpunkt + Alter + ROP + Geschlecht + TS_v_V + Gruppe*Zeitpunkt + (1 | Zeitpunkt), data = data_real_filter)

summary(real_KOOS_all_diff)

summary(real_KOOS_Kovariate_diff)

means_KOOS_real_gesamt <- tapply(data_real_filter$KOOS_gesamt, list(data_real_filter$Gruppe, data_real_filter$Zeitpunkt), mean)

means_KOOS_real_gesamt

sd_KOOS_real_gesamt <- tapply(data_real_filter$KOOS_gesamt, list(data_real_filter$Gruppe, data_real_filter$Zeitpunkt), sd)

sd_KOOS_real_gesamt

n_KOOS_real <- sum(!is.na(data_real_filter$KOOS_gesamt))

n_KOOS_real

t_value_KOOS_real <- qt(0.975, df = n_KOOS_real - 1)

t_value_KOOS_real

stderr_KOOS_real <- sd_KOOS_real_gesamt / sqrt(n_KOOS_real)

untere_grenze_KOOS_real <- means_KOOS_real_gesamt - t_value_KOOS_real * stderr_KOOS_real

obere_grenze_KOOS_real <- means_KOOS_real_gesamt + t_value_KOOS_real * stderr_KOOS_real

konfidenzintervalle_KOOS_real <- data.frame(

untere_grenze = untere_grenze_KOOS_real,

obere_grenze = obere_grenze_KOOS_real

)

print(konfidenzintervalle_KOOS_real)

data_aggregated_KOOS_real <- data_real_filter %>%

filter(!is.na(Gruppe)) %>% # NA-Werte in Gruppe entfernen

group_by(Zeitpunkt, Gruppe) %>%

summarise(

Mittelwert_KOOS_real_gesamt = mean(KOOS_gesamt),

SD_KOOS_real_gesamt = sd(KOOS_gesamt),

.groups = "drop"

)

data_aggregated_KOOS_real <- data_aggregated_KOOS_real %>%

arrange(Gruppe, Zeitpunkt) %>% # Sicherstellen, dass die Daten sortiert sind

mutate(Group_Lines = cumsum(lag(Zeitpunkt, default = first(Zeitpunkt)) != Zeitpunkt - 1))

ggplot(data_aggregated_KOOS_real, aes(x = Zeitpunkt, y = Mittelwert_KOOS_real_gesamt, color = as.factor(Gruppe))) +

geom_point(position = position_dodge(width = 0.2), size = 3) + # Punkte leicht versetzt

geom_line(aes(group = interaction(Gruppe, Group_Lines)), position = position_dodge(width = 0.2), size = 1) + # Linien leicht versetzt, ohne Verbindung 2-4

geom_errorbar(aes(

ymin = Mittelwert_KOOS_real_gesamt - (t_value_KOOS_real * SD_KOOS_real_gesamt / sqrt(n_KOOS_real)),

ymax = Mittelwert_KOOS_real_gesamt + (t_value_KOOS_real * SD_KOOS_real_gesamt / sqrt(n_KOOS_real))

),

width = 0.2, size = 0.5, position = position_dodge(width = 0.2)) + # Fehlerbalken leicht versetzt

scale_color_manual(values = c("blue", "red"), labels = c("Comparator", "Intervention")) +

scale_y_continuous(limits = c(40, 80), breaks = seq(0, 100, 10)) +

scale_x_continuous(breaks = 1:7) +

labs(

title = "KOOS sum score per protocol",

x = "time",

y = "KOOS sum score without imputation",

color = "group"

) +

theme_minimal()

imput_KOOS_all_diff <- lmer(KOOS_Diff ~ Gruppe + Zeitpunkt + (1|Zeitpunkt) + KOOS_Baseline*Gruppe*as.factor(Zeitpunkt), data = data_imput)

imput_KOOS_Kovariate_diff <- lmer(KOOS_Diff ~ KOOS_Baseline + Gruppe + Zeitpunkt + Alter + ROP + Geschlecht + TS_v_V + Gruppe*Zeitpunkt + (1 | Zeitpunkt), data = data_imput)

summary(imput_KOOS_all_diff)

summary(imput_KOOS_Kovariate_diff)

means_KOOS_gesamt <- tapply(data_imput$KOOS_gesamt, list(data_imput$Gruppe, data_imput$Zeitpunkt), mean)

means_KOOS_gesamt

sd_KOOS_gesamt <- tapply(data_imput$KOOS_gesamt, list(data_imput$Gruppe, data_imput$Zeitpunkt), sd)

sd_KOOS_gesamt

n_KOOS_gs <- sum(!is.na(data_imput$KOOS_gesamt))

n_KOOS_gs

t_value_KOOS_gs <- qt(0.975, df = n_KOOS_gs - 1)

t_value_KOOS_gs

stderr_KOOS_gesamt <- sd_KOOS_gesamt / sqrt(n_KOOS_gs)

untere_grenze_KOOS_gesamt <- means_KOOS_gesamt - t_value_KOOS_gs * stderr_KOOS_gesamt

obere_grenze_KOOS_gesamt <- means_KOOS_gesamt + t_value_KOOS_gs * stderr_KOOS_gesamt

konfidenzintervalle_KOOS_gesamt <- data.frame(

untere_grenze = untere_grenze_KOOS_gesamt,

obere_grenze = obere_grenze_KOOS_gesamt

)

print(konfidenzintervalle_KOOS_gesamt)

# A.8 Per-Protocol Analysis

In the following per-protocol analysis only the particpant’s actual data were included with no imputation of missing values.

Table A.8.1 outcomes details KOOS sum score with no imputation of missing values. (SD=standard deviation, CI=confidence interval, t1=during the initial hospital anamnesis, t2=1-7 days prior to ACL reconstruction, t3=on the day of surgery, t4= 30 days postoperatively, t5= 60 days postoperatively, t6= 90 days postoperatively and t7=180 days postoperatively)

| **group** | **time point** | **mean** | **SD** | **CI** | |
| --- | --- | --- | --- | --- | --- |
| **comparator group** | **t1** | 50.485 | 13.505 | 49.11- | 51.86 |
|  | **t2** | 60.918 | 11.207 | 59.777- | 62.059 |
|  | **t4** | 47.912 | 11.045 | 46.787- | 49.036 |
|  | **t5** | 60.731 | 12.079 | 59.501- | 61.961 |
|  | **t6** | 68.235 | 11.73 | 67.041- | 69.429 |
|  | **t7** | 73.027 | 14.919 | 71.508- | 74.546 |
| **intervention group** | **t1** | 44.846 | 14.501 | 43.369- | 46.322 |
|  | **t2** | 61.763 | 13.956 | 60.342- | 63.184 |
|  | **t4** | 44.907 | 12.343 | 43.65- | 46.164 |
|  | **t5** | 60.115 | 12.843 | 58.808- | 61.423 |
|  | **t6** | 67.533 | 12.759 | 66.234- | 68.832 |
|  | **t7** | 71.801 | 11.969 | 70.582- | 73.02 |

Table A.8.2 Primary outcomes of the mixed model analyses with repeated measures: KOOS sum score with no imputation of missing values (std.error= standard error, df = degrees of freedom, Diff = Difference between time points)

| **fixed effects** | **estimate** | **std.error** | **t value** | **df** | **p value** | **Significance** |
| --- | --- | --- | --- | --- | --- | --- |
| (Intercept) | 24.87742 | 15.77507 | 1.577009 | <0.001 | 1 |  |
| group | 16.66056 | 10.25413 | 1.624767 | 269.9991 | 0.105379 |  |
| time point | 3.994953 | 3.635796 | 1.098784 | <0.001 | 1 |  |
| KOOS sum score Baseline | -0.44715 | 0.157755 | -2.83445 | 269.9988 | 0.004937 | ** |
| as.factor(time point) Diff Baseline-4 | -12.5162 | 14.07755 | -0.88909 | <0.001 | 1 |  |
| as.factor(time point) Diff Baseline-5 | 0.3103 | 14.86174 | 0.020879 | <0.001 | 1 |  |
| as.factor(time point) Diff Baseline-6 | 13.63805 | 17.09567 | 0.797749 | <0.001 | 1 |  |
| group:KOOS sum score Baseline | -0.27859 | 0.204143 | -1.3647 | 269.9992 | 0.173484 |  |
| group:as.factor(time point) Diff Baseline-4 | -16.2512 | 15.01671 | -1.08221 | 269.9998 | 0.280126 |  |
| group:as.factor(time point) Diff Baseline-5 | -16.9385 | 15.1037 | -1.12148 | 269.9998 | 0.263081 |  |
| group:as.factor(time point) Diff Baseline-6 | -32.987 | 15.89564 | -2.07522 | 269.9998 | 0.038913 | * |
| group:as.factor(time point) Diff Baseline-7 | -14.7473 | 18.12554 | -0.81362 | 269.9997 | 0.416581 |  |
| group:KOOS Baseline:as.factor(time point) Diff Baseline-4 | 0.257939 | 0.293954 | 0.877479 | 269.9998 | 0.381007 |  |
| group:KOOS Baseline:as.factor(time point) Diff Baseline-5 | 0.310511 | 0.295252 | 1.05168 | 269.9998 | 0.293887 |  |
| group:KOOS Baseline:as.factor(time point) Diff Baseline-6 | 0.631243 | 0.30477 | 2.071208 | 269.9998 | 0.039289 | * |
| group:KOOS Baseline:as.factor(time point) Diff Baseline-7 | 0.277997 | 0.340813 | 0.815685 | 269.9997 | 0.415399 |  |

Table A.8.3 fixed effects KOOS sum score with no imputation of missing values (std.error= standard error, df = degrees of freedom)

|  | **estimate** | **std.error** | **t value** | **df** | **p value** | **Significance** |
| --- | --- | --- | --- | --- | --- | --- |
| (Intercept) | 32.37222206 | 13.71613366 | 2.360156504 | 4.444260766 | 0.071172659 |  |
| KOOS sum score Baseline | -0.659937145 | 0.047378231 | -13.92912168 | 278.0128712 | 8.16075E-34 | *** |
| group | 1.927466683 | 3.785289617 | 0.509199263 | 278.0949821 | 0.611016346 |  |
| time point | 2.729443011 | 2.480637709 | 1.100298928 | 3.215927762 | 0.346670928 |  |
| age | -0.148166937 | 0.07135319 | -2.076528575 | 278.0151611 | 0.038763658 | * |
| time between rupture and surgery | 0.037554761 | 0.017812797 | 2.108302328 | 278.0127715 | 0.035899624 | * |
| gender | -0.845724249 | 1.447013648 | -0.584461833 | 278.0235616 | 0.559383591 |  |
| Tegner Score before injury | 0.200581903 | 0.542936403 | 0.36943904 | 278.0170967 | 0.712081732 |  |
| group*time point | -0.025068686 | 0.776230762 | -0.032295404 | 278.1110636 | 0.97425964 |  |


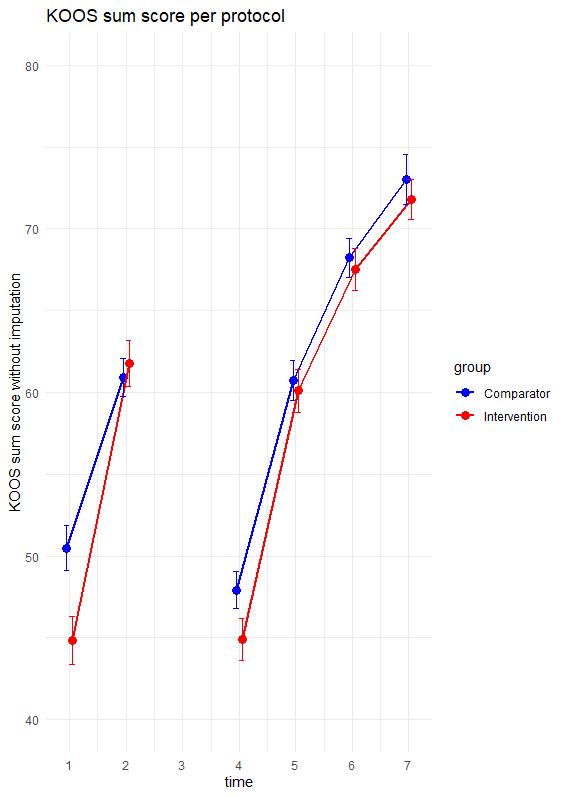


Figure A.8 Means and 95% confidence intervals (CI) of the KOOS sum score (per protocol), separated by groups. The x-axis shows the 7 measurement time points, (t1=during the initial hospital anamnesis, t2=1-7 days prior to ACL reconstruction, t3=on the day of surgery, t4= 30 days postoperatively, t5= 60 days postoperatively, t6= 90 days postoperatively and t7=180 days postoperatively), the y-axis shows the values of the KOOS sum score (0-100%) and 95% CI; (KOOS = Knee Injury and Osteoarthritis Outcome Score).

# A.9 Mixed Models outcomes – KOOS sum Score

Table A.9.1 outcomes details KOOS sum score (SD=standard deviation, CI=confidence interval, t1=during the initial hospital anamnesis, t2=1-7 days prior to ACL reconstruction, t3=on the day of surgery, t4= 30 days postoperatively, t5= 60 days postoperatively, t6= 90 days postoperatively and t7=180 days postoperatively)

| **group** | **time point** | **Mean** | **SD** | **CI** | |
| --- | --- | --- | --- | --- | --- |
| **comparator group** | **1** | 51.006 | 11.88 | 50.095- | 51.917 |
|  | **2** | 59.182 | 10.184 | 58.401- | 59.962 |
|  | **4** | 52.419 | 10.947 | 51.58- | 53.258 |
|  | **5** | 60.473 | 9.3335 | 59.757- | 61.188 |
|  | **6** | 66.149 | 8.908 | 65.466- | 66.832 |
|  | **7** | 68.375 | 10.253 | 67.589- | 69.161 |
| **intervention group** | **1** | 46.044 | 12.754 | 45.066- | 47.021 |
|  | **2** | 58.523 | 13.258 | 57.507- | 59.54 |
|  | **4** | 47.324 | 11.378 | 46.451- | 48.196 |
|  | **5** | 58.929 | 10.845 | 58.098- | 59.76 |
|  | **6** | 64.803 | 10.321 | 64.012- | 65.594 |
|  | **7** | 67.513 | 10.999 | 66.669- | 68.356 |

Table A.9.2 Primary outcomes of the mixed model analyses with repeated measures: KOOS sum score (std.error= standard error, df = degrees of freedom, Diff = Difference between time points)

|  | **estimate** | **std.error** | **t value** | **df** | **p value** | **Significance** |
| --- | --- | --- | --- | --- | --- | --- |
| (Intercept) | 33.10704 | 11.09962 | 2.982719 | <0.001 | 1 |  |
| group | 8.903359 | 7.96829 | 1.117349 | 521.9999 | 0.264359 |  |
| time point | 3.674642 | 2.167548 | 1.695299 | <0.001 | 1 |  |
| KOOS sum score Baseline | -0.63288 | 0.116006 | -5.4556 | 522 | <0.001 | *** |
| as.factor(time point) Diff Baseline-4 | -3.00812 | 9.478706 | -0.31736 | <0.001 | 1 |  |
| as.factor(time point) Diff Baseline-5 | 3.184566 | 9.493962 | 0.335431 | <0.001 | 1 |  |
| as.factor(time point) Diff Baseline-6 | 8.230274 | 9.99106 | 0.823764 | <0.001 | 1 |  |
| group:KOOS sum score Baseline | -0.16809 | 0.158532 | -1.06032 | 521.9998 | 0.289488 |  |
| group:as.factor(time point) Diff Baseline-4 | -23.4992 | 11.35977 | -2.06863 | 521.9999 | 0.039073 | * |
| group:as.factor(time point) Diff Baseline-5 | -17.1104 | 11.35977 | -1.50622 | 522 | 0.132614 |  |
| group:as.factor(time point) Diff Baseline-6 | -23.5058 | 11.35977 | -2.06921 | 522 | 0.039018 | * |
| group:as.factor(time point) Diff Baseline-7 | -13.7886 | 11.35977 | -1.21381 | 521.9998 | 0.225368 |  |
| group:KOOS sum score Baseline:as.factor(time point) Diff Baseline-4 | 0.389534 | 0.22547 | 1.727654 | 521.9999 | 0.084642 |  |
| group:KOOS sum score Baseline:as.factor(time point) Diff Baseline-5 | 0.324365 | 0.22547 | 1.438616 | 521.9999 | 0.150859 |  |
| group:KOOS sum score Baseline:as.factor(time point) Diff Baseline-6 | 0.460658 | 0.22547 | 2.043101 | 522 | 0.041544 | * |
| group:KOOS sum score Baseline:as.factor(time point) Diff Baseline-7 | 0.275039 | 0.22547 | 1.219845 | 521.9998 | 0.223074 |  |

Table A.9.3 fixed effects KOOS sum score (std.error= standard error, df = degrees of freedom)

|  | **estimate** | **std.error** | **t value** | **df** | **p.value** | **Significance** |
| --- | --- | --- | --- | --- | --- | --- |
| (Intercept) | 42.53819 | 10.1864 | 4.17598 | 4.280788 | 0.012109 | * |
| KOOS sum score Baseline | -0.72663 | 0.038248 | -18.9978 | 475.0016 | <0.001 | *** |
| group | -0.95367 | 2.768251 | -0.3445 | 475.0051 | 0.730621 |  |
| time point | 2.284103 | 1.847679 | 1.236201 | 3.133262 | 0.300965 |  |
| age | -0.13371 | 0.050749 | -2.63476 | 475.0016 | 0.008695 | ** |
| time between rupture and surgery | -0.00077 | 0.010988 | -0.06992 | 475.0017 | 0.944285 |  |
| gender | -0.46723 | 1.055955 | -0.44247 | 475.0014 | 0.658348 |  |
| Tegner Score before injury | -0.20687 | 0.380457 | -0.54375 | 475.0027 | 0.586868 |  |
| group*time point | 0.206027 | 0.544659 | 0.378269 | 475.0041 | 0.7054 |  |

# A. 10. Mixed models outcomes – KOOS subscales

# Subscale Symptoms

Table A.10.1.1 outcomes details KOOS subscale symptoms (SD=standard deviation, CI=confidence interval, t1=during the initial hospital anamnesis, t2=1-7 days prior to ACL reconstruction, t3=on the day of surgery, t4= 30 days postoperatively, t5= 60 days postoperatively, t6= 90 days postoperatively and t7=180 days postoperatively)

| **group** | **time point** | **Mean** | **SD** | **CI** | |
| --- | --- | --- | --- | --- | --- |
| **comparator group** | **t1** | 57.206 | 12.307 | 56.262- | 58.149 |
|  | **t2** | 60.15 | 12.107 | 59.222- | 61.079 |
|  | **t4** | 57.624 | 11.206 | 56.765- | 58.483 |
|  | **t5** | 58.31 | 10.364 | 57.516- | 59.105 |
|  | **t6** | 61.332 | 10.07 | 60.56- | 62.104 |
|  | **t7** | 60.577 | 9.5403 | 59.846- | 61.308 |
| **intervention group** | **t1** | 56.892 | 11.451 | 56.014- | 57.77 |
|  | **t2** | 60.338 | 12.421 | 59.386- | 61.291 |
|  | **t4** | 55.974 | 12.43 | 55.021- | 56.927 |
|  | **t5** | 59.935 | 12.871 | 58.948- | 60.922 |
|  | **t6** | 60.779 | 12.457 | 59.824- | 61.734 |
|  | **t7** | 59.351 | 10.659 | 58.533- | 60.168 |

Table A.10.1.2 Primary outcomes of the mixed model analyses with repeated measures: KOOS subscale symptoms (std.error= standard error, df = degrees of freedom, Diff = Difference between time points)

|  | **estimate** | **std.error** | **t value** | **df** | **p.value** | **Significance** |
| --- | --- | --- | --- | --- | --- | --- |
| (Intercept) | 25.55653 | 10.85468 | 2.354424 | 522 | 0.018921 | * |
| group | 23.19787 | 10.55625 | 2.197548 | 522 | 0.02842 | * |
| time point | 4.429304 | 2.125369 | 2.084017 | 522 | 0.037644 | * |
| KOOS subscale symptoms Baseline | -0.55013 | 0.123478 | -4.45524 | 522 | <0.001 | *** |
| as.factor(time point)Diff Baseline-4 | 3.282254 | 9.309288 | 0.352578 | 522 | 0.724547 |  |
| as.factor(time point)Diff Baseline-5 | 9.389813 | 9.331716 | 1.006226 | 522 | 0.314773 |  |
| as.factor(time point)Diff Baseline-6 | 9.795169 | 9.825142 | 0.996949 | 522 | 0.319251 |  |
| group: KOOS subscale symptoms Baseline | -0.40197 | 0.181267 | -2.21755 | 522 | 0.027015 | * |
| group:as.factor(time point)Diff Baseline-4 | -10.0318 | 15.01363 | -0.66818 | 522 | 0.504314 |  |
| group:as.factor(time point)Diff Baseline-5 | -11.7822 | 15.01363 | -0.78477 | 522 | 0.432945 |  |
| group:as.factor(time point)Diff Baseline-6 | -30.43 | 15.01363 | -2.02682 | 522 | 0.043188 | * |
| group:as.factor(time point)Diff Baseline-7 | -5.87842 | 15.01363 | -0.39154 | 522 | 0.695559 |  |
| group:KOOS subscale symptoms Baseline: factor(time point) Diff Baseline-4 | 0.143248 | 0.257548 | 0.556198 | 522 | 0.578314 |  |
| group:KOOS subscale symptoms Baseline: factor(time point) Diff Baseline-5 | 0.229872 | 0.257548 | 0.892541 | 522 | 0.372514 |  |
| group:KOOS subscale symptoms Baseline: factor(time point) Diff Baseline-6 | 0.519409 | 0.257548 | 2.016748 | 522 | 0.044233 | * |
| group:KOOS subscale symptoms Baseline: factor(time point) Diff Baseline-7 | 0.076303 | 0.257548 | 0.296267 | 522 | 0.767144 |  |

Table A.10.1.3 fixed effects KOOS subscale symptoms (std.error= standard error, df = degrees of freedom)

|  | **estimate** | **std.error** | **t value** | **df** | **p.value** | **Significance** |
| --- | --- | --- | --- | --- | --- | --- |
| (Intercept) | 51.30301 | 5.815385 | 8.821945 | 43.34862 | <0.001 | *** |
| KOOS subscale symptoms Baseline | -0.93701 | 0.04535 | -20.6619 | 475.0139 | <0.001 | *** |
| group | 1.363224 | 3.082658 | 0.442224 | 475.0577 | 0.658529 |  |
| time point | 0.25366 | 0.651584 | 0.389298 | 4.861607 | 0.713522 |  |
| age | -0.02678 | 0.05659 | -0.4732 | 475.0162 | 0.636287 |  |
| time between rupture and surgery | 0.019374 | 0.012209 | 1.586854 | 475.0166 | 0.113211 |  |
| gender | -0.45141 | 1.183014 | -0.38157 | 475.0135 | 0.702949 |  |
| Tegner Score before injury | 0.491102 | 0.424868 | 1.155893 | 475.0298 | 0.248306 |  |
| group*time point | -0.27603 | 0.60786 | -0.4541 | 475.047 | 0.649965 |  |

# Subscale Pain

Table A.10.2.1 outcomes details KOOS subscale pain (SD=standard deviation, CI=confidence interval, t1=during the initial hospital anamnesis, t2=1-7 days prior to ACL reconstruction, t3=on the day of surgery, t4= 30 days postoperatively, t5= 60 days postoperatively, t6= 90 days postoperatively and t7=180 days postoperatively)

| **group** | **time point** | **Mean** | **SD** | **CI** | |
| --- | --- | --- | --- | --- | --- |
| **comparator group** | **t1** | 61.501 | 13.491 | 60.467- | 62.535 |
|  | **t2** | 70.809 | 15.679 | 69.607- | 72.011 |
|  | **t4** | 64.316 | 13.252 | 63.3- | 65.332 |
|  | **t5** | 72.596 | 10.85 | 71.764- | 73.428 |
|  | **t6** | 77.938 | 10.149 | 77.16- | 78.716 |
|  | **t7** | 80.395 | 10.014 | 79.628- | 81.163 |
| **intervention group** | **t1** | 59.357 | 15.382 | 58.177- | 60.536 |
|  | **t2** | 70.712 | 17.403 | 69.377- | 72.046 |
|  | **t4** | 61.919 | 14.08 | 60.84- | 62.999 |
|  | **t5** | 72.929 | 12.028 | 72.007- | 73.851 |
|  | **t6** | 78.485 | 10.932 | 77.647- | 79.323 |
|  | **t7** | 77.778 | 15.466 | 76.592- | 78.963 |

Table A.10.2.2 Primary outcomes of the mixed model analyses with repeated measures: KOOS subscale pain (std.error= standard error, df = degrees of freedom, Diff = Difference between time points)

|  | **estimate** | **std.error** | **t value** | **df** | **p.value** | **Significance** |
| --- | --- | --- | --- | --- | --- | --- |
| (Intercept) | 34.89176 | 11.96544 | 2.916044 | 522 | 0.003697 | ** |
| group | 14.77603 | 10.70266 | 1.380595 | 522 | 0.167994 |  |
| time point | 5.571823 | 2.34005 | 2.38107 | 522 | 0.01762 | * |
| KOOS subscale pain Baseline | -0.59718 | 0.129315 | -4.61807 | 522 | 4.88E-06 | *** |
| as.factor(time point)Diff Baseline-4 | -1.67843 | 10.24217 | -0.16387 | 522 | 0.869893 |  |
| as.factor(time point)Diff Baseline-5 | 2.804617 | 10.26317 | 0.27327 | 522 | 0.784754 |  |
| as.factor(time point)Diff Baseline-6 | 5.540333 | 10.80347 | 0.512829 | 522 | 0.608288 |  |
| group: KOOS subscale pain Baseline | -0.23603 | 0.172002 | -1.37223 | 522 | 0.17058 |  |
| group:as.factor(time point)Diff Baseline-4 | -29.1677 | 15.22071 | -1.91632 | 522 | 0.055871 |  |
| group:as.factor(time point)Diff Baseline-5 | -14.8382 | 15.22071 | -0.97487 | 522 | 0.330078 |  |
| group:as.factor(time point)Diff Baseline-6 | -15.6109 | 15.22071 | -1.02563 | 522 | 0.305539 |  |
| group:as.factor(time point)Diff Baseline-7 | -22.8377 | 15.22071 | -1.50044 | 522 | 0.134106 |  |
| group:KOOS subscale pain Baseline: factor(time point) Diff Baseline-4 | 0.442055 | 0.244521 | 1.807837 | 522 | 0.071207 |  |
| group:KOOS subscale pain Baseline: factor(time point) Diff Baseline-5 | 0.246201 | 0.244521 | 1.00687 | 522 | 0.314464 |  |
| group:KOOS subscale pain Baseline: factor(time point) Diff Baseline-6 | 0.261293 | 0.244521 | 1.068591 | 522 | 0.285748 |  |
| group:KOOS subscale pain Baseline: factor(time point) Diff Baseline-7 | 0.330793 | 0.244521 | 1.352819 | 522 | 0.176699 |  |

Table A.10.2.3 fixed effects KOOS subscale pain (std.error= standard error, df = degrees of freedom)

|  | **estimate** | **std.error** | **t value** | **df** | **p.value** | **Significance** |
| --- | --- | --- | --- | --- | --- | --- |
| (Intercept) | 55.61786 | 10.02997 | 5.545167 | 5.893491 | 0.001541 | ** |
| KOOS subscale pain Baseline | -0.81721 | 0.0407 | -20.079 | 475.0023 | <0.001 | *** |
| group | 1.430758 | 3.498308 | 0.408986 | 475.0091 | 0.682734 |  |
| time point | 2.395266 | 1.696053 | 1.412258 | 3.260749 | 0.245821 |  |
| age | -0.17408 | 0.064208 | -2.71119 | 475.0027 | 0.006947 | ** |
| time between rupture and surgery | -0.00353 | 0.013817 | -0.25525 | 475.0028 | 0.798644 |  |
| gender | 0.907435 | 1.342837 | 0.67576 | 475.0023 | 0.499522 |  |
| Tegner Score before injury | -0.12144 | 0.484652 | -0.25056 | 475.0048 | 0.80226 |  |
| group*time point | -0.27324 | 0.689594 | -0.39623 | 475.0075 | 0.692114 |  |

# Subscale activity

Table A.10.3.1 outcomes details KOOS subscale activity (SD=standard deviation, CI=confidence interval, t1=during the initial hospital anamnesis, t2=1-7 days prior to ACL reconstruction, t3=on the day of surgery, t4= 30 days postoperatively, t5= 60 days postoperatively, t6= 90 days postoperatively and t7=180 days postoperatively)

| **group** | **time point** | **Mean** | **SD** | **CI** | |
| --- | --- | --- | --- | --- | --- |
| **comparator group** | **t1** | 69.7884417 | 15.6302702 | 68.5901408- | 70.9867426 |
|  | **t2** | 81.0887513 | 13.1311219 | 80.0820486- | 82.095454 |
|  | **t4** | 70.418552 | 14.140378 | 69.3344743- | 71.5026298 |
|  | **t5** | 80.7126697 | 10.3481604 | 79.9193238- | 81.5060156 |
|  | **t6** | 86.2839367 | 8.54382286 | 85.628921- | 86.9389523 |
|  | **t7** | 86.1425339 | 8.62594104 | 85.4812227- | 86.8038452 |
| **intervention group** | **t1** | 64.4736842 | 17.061335 | 63.1656701- | 65.7816983 |
|  | **t2** | 81.501548 | 14.0641147 | 80.423317- | 82.579779 |
|  | **t4** | 66.9518717 | 16.499679 | 65.6869171- | 68.2168262 |
|  | **t5** | 82.3262032 | 10.5344301 | 81.5185769- | 83.1338295 |
|  | **t6** | 87.4331551 | 9.59256731 | 86.6977371- | 88.1685731 |
|  | **t7** | 89.4117647 | 9.2550612 | 88.7022217- | 90.1213077 |

Table A.10.3.2 Primary outcomes of the mixed model analyses with repeated measures: KOOS subscale activity (std.error= standard error, df = degrees of freedom, Diff = Difference between time points)

|  | **estimate** | **std.error** | **t value** | **df** | **p.value** | **Signifi**  **cance** |
| --- | --- | --- | --- | --- | --- | --- |
| (Intercept) | 44.57762 | 11.26564 | 3.956953 | 5.09E-08 | 0.999999 |  |
| group | 24.43739 | 9.076295 | 2.692441 | 521.9994 | 0.007321 | ** |
| time point | 4.221235 | 2.194756 | 1.923327 | 5.15E-08 | 0.999999 |  |
| KOOS subscale activity Baseline | -0.5978 | 0.096539 | -6.19237 | 521.9983 | <0.001 | *** |
| as.factor(time point)Diff Baseline-4 | -13.0887 | 9.58377 | -1.36572 | 5.18E-08 | 1 |  |
| as.factor(time point)Diff Baseline-5 | -4.89401 | 9.592283 | -0.5102 | 5.2E-08 | 1 |  |
| as.factor(time point)Diff Baseline-6 | 5.339797 | 10.09005 | 0.529214 | 5.21E-08 | 1 |  |
| group: KOOS subscale activity Baseline | -0.33947 | 0.130926 | -2.59286 | 521.9994 | 0.009785 | ** |
| group:as.factor(time point)Diff Baseline-4 | -28.5435 | 12.88704 | -2.2149 | 521.9997 | 0.027198 | * |
| group:as.factor(time point)Diff Baseline-5 | -5.43997 | 12.88704 | -0.42213 | 521.9999 | 0.673106 |  |
| group:as.factor(time point)Diff Baseline-6 | -15.0944 | 12.88704 | -1.17129 | 521.9999 | 0.242018 |  |
| group:as.factor(time point)Diff Baseline-7 | -14.9108 | 12.88704 | -1.15704 | 521.9997 | 0.247787 |  |
| group:KOOS subscale activity Baseline: factor(time point) Diff Baseline-4 | 0.374603 | 0.18558 | 2.018556 | 521.9997 | 0.044044 | * |
| group:KOOS subscale activity Baseline: factor(time point) Diff Baseline-5 | 0.093516 | 0.18558 | 0.50391 | 521.9999 | 0.614537 |  |
| group:KOOS subscale activity Baseline: factor(time point) Diff Baseline-6 | 0.22543 | 0.18558 | 1.214735 | 521.9998 | 0.225016 |  |
| group:KOOS subscale activity Baseline: factor(time point) Diff Baseline-7 | 0.256487 | 0.18558 | 1.382085 | 521.9997 | 0.167537 |  |

Table A.10.3.3 fixed effects KOOS subscale activity (std.error= standard error, df = degrees of freedom)

|  | **estimate** | **std.error** | **t value** | **df** | **p.value** | **Significance** |
| --- | --- | --- | --- | --- | --- | --- |
| (Intercept) | 71.27009 | 12.00158 | 5.938392 | 4.090405 | 0.003756 | ** |
| KOOS subscale activity Baseline | -0.80996 | 0.030882 | -26.2279 | 475.0011 | <0.001 | *** |
| group | -1.28123 | 3.007033 | -0.42608 | 475.004 | 0.670244 |  |
| time point | 1.748111 | 2.197856 | 0.795371 | 3.110517 | 0.482623 |  |
| age | -0.24551 | 0.055254 | -4.44336 | 475.0012 | <0.001 | *** |
| time between rupture and surgery | -0.00201 | 0.012038 | -0.16733 | 475.0013 | 0.867183 |  |
| gender | -2.08188 | 1.148525 | -1.81266 | 475.001 | 0.070516 |  |
| Tegner Score before injury | -0.13899 | 0.413848 | -0.33586 | 475.0021 | 0.737125 |  |
| group*time point | 0.72467 | 0.592188 | 1.223717 | 475.0032 | 0.221666 |  |

# Subscale sports

Table A.10.4.1 outcomes details KOOS subscale sports (SD=standard deviation, CI=confidence interval, t1=during the initial hospital anamnesis, t2=1-7 days prior to ACL reconstruction, t3=on the day of surgery, t4= 30 days postoperatively, t5= 60 days postoperatively, t6= 90 days postoperatively and t7=180 days postoperatively)

| **group** | **time point** | **Mean** | **SD** | **CI** | |
| --- | --- | --- | --- | --- | --- |
| **comparator group** | **t1** | 34.2982456 | 19.7628484 | 32.7831189- | 35.8133723 |
|  | **t2** | 47.4561404 | 19.9356735 | 45.9277639- | 48.9845168 |
|  | **t4** | 34.0384615 | 21.3752299 | 32.399721- | 35.6772021 |
|  | **t5** | 48.5576923 | 20.0327315 | 47.0218749- | 50.0935097 |
|  | **t6** | 57.5961538 | 17.6148997 | 56.2457005- | 58.9466072 |
|  | **t7** | 61.1538462 | 19.7934279 | 59.6363751- | 62.6713173 |
| **intervention group** | **t1** | 23.9473684 | 20.4358063 | 22.3806491- | 25.5140877 |
|  | **t2** | 43.7719298 | 23.4764453 | 41.9720987- | 45.5717609 |
|  | **t4** | 22 | 19.7343468 | 20.4870584- | 23.5129416 |
|  | **t5** | 39 | 20.7364414 | 37.4102324- | 40.5897676 |
|  | **t6** | 51.6363636 | 19.9084605 | 50.1100735- | 53.1626538 |
|  | **t7** | 62.7272727 | 19.5746005 | 61.2265781- | 64.2279673 |

Table A.10.4.2 Primary outcomes of the mixed model analyses with repeated measures: KOOS subscale sports (std.error= standard error, df = degrees of freedom, Diff = Difference between time points)

|  | **estimate** | **std.error** | **t value** | **df** | **p.value** | **Signifi**  **cance** |
| --- | --- | --- | --- | --- | --- | --- |
| (Intercept) | 32.5358 | 15.31239 | 2.124802 | 522 | 0.034072 | * |
| group | -0.66888 | 6.6335 | -0.10083 | 522 | 0.919721 |  |
| time point | 2.815933 | 2.987302 | 0.942634 | 522 | 0.346304 |  |
| KOOS subscale sports Baseline | -0.72919 | 0.1332 | -5.47435 | 522 | <0.001 | *** |
| as.factor(time point)Diff Baseline-4 | -16.0306 | 13.05571 | -1.22786 | 522 | 0.220053 |  |
| as.factor(time point)Diff Baseline-5 | -3.52335 | 13.07285 | -0.26952 | 522 | 0.787639 |  |
| as.factor(time point)Diff Baseline-6 | 4.265352 | 13.75483 | 0.310098 | 522 | 0.75661 |  |
| group: KOOS subscale sports Baseline | -0.00886 | 0.185298 | -0.04781 | 522 | 0.961885 |  |
| group:as.factor(time point)Diff Baseline-4 | -15.0132 | 9.529504 | -1.57545 | 522 | 0.115759 |  |
| group:as.factor(time point)Diff Baseline-5 | -12.4687 | 9.529504 | -1.30843 | 522 | 0.191304 |  |
| group:as.factor(time point)Diff Baseline-6 | -8.81383 | 9.529504 | -0.9249 | 522 | 0.355445 |  |
| group:as.factor(time point)Diff Baseline-7 | 5.74642 | 9.529504 | 0.603014 | 522 | 0.546762 |  |
| group:KOOS subscale sports Baseline: factor(time point) Diff Baseline-4 | 0.239732 | 0.264416 | 0.906647 | 522 | 0.365012 |  |
| group:KOOS subscale sports Baseline: factor(time point) Diff Baseline-5 | 0.226873 | 0.264416 | 0.858016 | 522 | 0.391277 |  |
| group:KOOS subscale sports Baseline: factor(time point) Diff Baseline-6 | 0.204619 | 0.264416 | 0.773851 | 522 | 0.439369 |  |
| group:KOOS subscale sports Baseline: factor(time point) Diff Baseline-7 | -0.02348 | 0.264416 | -0.08879 | 522 | 0.929286 |  |

Table A.10.4.3 fixed effects KOOS subscale sports (std.error= standard error, df = degrees of freedom)

|  | **estimate** | **std.error** | **t value** | **df** | **p.value** | **Significance** |
| --- | --- | --- | --- | --- | --- | --- |
| (Intercept) | 38.19701 | 18.79928 | 2.031834 | 4.077319 | 0.110648 |  |
| KOOS subscale sports Baseline | -0.69732 | 0.043962 | -15.8617 | 475.0019 | <0.001 | *** |
| group | -7.93165 | 5.230683 | -1.51637 | 475.0054 | 0.130091 |  |
| time point | 3.560101 | 3.452574 | 1.031144 | 3.136192 | 0.375334 |  |
| age | -0.30724 | 0.095815 | -3.20659 | 475.0018 | 0.001434 | ** |
| time between rupture and surgery | -0.02459 | 0.020959 | -1.17316 | 475.002 | 0.241321 |  |
| gender | -1.85913 | 1.992315 | -0.93315 | 475.0016 | 0.351216 |  |
| Tegner Score before injury | -0.24567 | 0.718458 | -0.34194 | 475.003 | 0.732546 |  |
| group*time point | 1.254717 | 1.027797 | 1.220783 | 475.0044 | 0.222774 |  |

# Subscale quality of life

Table A.10.5.1 outcomes details KOOS subscale quality of life (SD=standard deviation, CI=confidence interval, t1=during the initial hospital anamnesis, t2=1-7 days prior to ACL reconstruction, t3=on the day of surgery, t4= 30 days postoperatively, t5= 60 days postoperatively, t6= 90 days postoperatively and t7=180 days postoperatively)

| **group** | **time point** | **Mean** | **SD** | **CI** | |
| --- | --- | --- | --- | --- | --- |
| **comparator group** | **t1** | 32.2368421 | 16.6950338 | 30.9569106- | 33.5167736 |
|  | **t2** | 36.4035088 | 13.7298354 | 35.3509054- | 37.4561121 |
|  | **t4** | 35.6971154 | 14.8179588 | 34.5610906- | 36.8331401 |
|  | **t5** | 42.1875 | 13.0911094 | 41.1838648- | 43.1911352 |
|  | **t6** | 47.5961538 | 14.1742616 | 46.5094784- | 48.6828293 |
|  | **t7** | 53.6057692 | 17.6058665 | 52.2560084- | 54.9555301 |
| **intervention group** | **t1** | 25.5482456 | 17.450532 | 24.2103936- | 26.8860977 |
|  | **t2** | 36.2938596 | 16.3423969 | 35.0409632- | 37.5467561 |
|  | **t4** | 29.7727273 | 14.9247552 | 28.6285149- | 30.9169396 |
|  | **t5** | 40.4545455 | 17.6717165 | 39.0997362- | 41.8093547 |
|  | **t6** | 45.6818182 | 19.6862223 | 44.172566- | 47.1910703 |
|  | **t7** | 48.2954545 | 19.1490916 | 46.8273817- | 49.7635273 |

Table A.10.5.2 Primary outcomes of the mixed model analyses with repeated measures: KOOS subscale quality of life (std.error= standard error, df = degrees of freedom, Diff = Difference between time points)

|  | **estimate** | **std.error** | **t value** | **df** | **p.value** | **Significance** |
| --- | --- | --- | --- | --- | --- | --- |
| (Intercept) | 18.83611 | 9.743379 | 1.933222 | <0.001 | 1 |  |
| group | 0.209239 | 5.598149 | 0.037377 | 522 | 0.970199 |  |
| time point | 4.645817 | 1.911032 | 2.431052 | <0.001 | 1 |  |
| KOOS subscale quality of life Baseline | -0.74328 | 0.119781 | -6.20534 | 522 | <0.001 | *** |
| as.factor(time point)Diff Baseline-4 | -2.33592 | 8.379089 | -0.27878 | <0.001 | 1 |  |
| as.factor(time point)Diff Baseline-5 | -1.61582 | 8.403528 | -0.19228 | <0.001 | 1 |  |
| as.factor(time point)Diff Baseline-6 | 0.185776 | 8.850623 | 0.02099 | <0.001 | 1 |  |
| group: KOOS subscale quality of life Baseline | 0.054728 | 0.16577 | 0.330142 | 522 | 0.741425 |  |
| group:as.factor(time point)Diff Baseline-4 | -15.9851 | 8.088518 | -1.97627 | 522 | 0.04865 | * |
| group:as.factor(time point)Diff Baseline-5 | -15.4159 | 8.088518 | -1.90589 | 522 | 0.057213 |  |
| group:as.factor(time point)Diff Baseline-6 | -20.3953 | 8.088518 | -2.52151 | 522 | 0.011982 | * |
| group:as.factor(time point)Diff Baseline-7 | -16.3854 | 8.088518 | -2.02576 | 522 | 0.043298 | * |
| group:KOOS subscale quality of life Baseline: factor(time point) Diff Baseline-4 | 0.328516 | 0.238115 | 1.379654 | 522 | 0.168284 |  |
| group:KOOS subscale quality of life Baseline: factor(time point) Diff Baseline-5 | 0.476271 | 0.238115 | 2.000171 | 522 | 0.045999 | * |
| group:KOOS subscale quality of life Baseline: factor(time point) Diff Baseline-6 | 0.652742 | 0.238115 | 2.741291 | 522 | 0.00633 | ** |
| group:KOOS subscale quality of life Baseline: factor(time point) Diff Baseline-7 | 0.379946 | 0.238115 | 1.595639 | 522 | 0.111175 |  |

Table A.10.5.3 fixed effects KOOS subscale quality of life (std.error= standard error, df = degrees of freedom)

|  | **estimate** | **std.error** | **statistic** | **df** | **p.value** | **Significance** |
| --- | --- | --- | --- | --- | --- | --- |
| (Intercept) | 18.7252 | 9.082773 | 2.061617 | 7.722005 | 0.074435 |  |
| KOOS subscale quality of life Baseline | -0.65353 | 0.044376 | -14.7271 | 475.0071 | <0.001 | *** |
| group | 1.774178 | 4.096732 | 0.433072 | 475.0193 | 0.665159 |  |
| time point | 3.449025 | 1.461522 | 2.359887 | 3.50762 | 0.086816 |  |
| age | 0.052055 | 0.075927 | 0.685596 | 475.006 | 0.493302 |  |
| time between rupture and surgery | 0.004356 | 0.016166 | 0.269482 | 475.0068 | 0.787676 |  |
| gender | 0.586186 | 1.56728 | 0.374015 | 475.0056 | 0.70856 |  |
| Tegner Score before injury | -1.03517 | 0.562774 | -1.83941 | 475.0102 | 0.066479 |  |
| group*time point | -0.39443 | 0.80558 | -0.48962 | 475.0155 | 0.624626 |  |

# A.11 Mixed Models Outcomes – ACL-RSI

Table A.11.1 outcomes details ACL-RSI (SD=standard deviation, CI=confidence interval, t1=during the initial hospital anamnesis, t2=1-7 days prior to ACL reconstruction, t3=on the day of surgery, t4= 30 days postoperatively, t5= 60 days postoperatively, t6= 90 days postoperatively and t7=180 days postoperatively)

| **group** | **time point** | **Mean** | **SD** | **CI** | |
| --- | --- | --- | --- | --- | --- |
| **comparator group** | **t1** | 51.2573099 | 18.7242699 | 49.9326888- | 52.5819311 |
|  | **t2** | 55.7748538 | 16.3806255 | 54.6160303- | 56.9336773 |
|  | **t3** | 56.2426901 | 14.7095919 | 55.2020814- | 57.2832987 |
|  | **t4** | 53.7660256 | 16.4632111 | 52.6013598- | 54.9306915 |
|  | **t5** | 53.5096154 | 14.5646113 | 52.4792632- | 54.5399676 |
|  | **t6** | 57.4519231 | 14.9357862 | 56.3953127- | 58.5085335 |
|  | **t7** | 55.9615385 | 14.6504149 | 54.9251162- | 56.9979607 |
| **intervention group** | **t1** | 53.5087719 | 17.6606408 | 52.2593956- | 54.7581482 |
|  | **t2** | 54.9269006 | 18.1895612 | 53.6401066- | 56.2136946 |
|  | **t3** | 54.8245614 | 17.4295185 | 53.5915355- | 56.0575873 |
|  | **t4** | 57 | 17.5686484 | 55.7571316- | 58.2428684 |
|  | **t5** | 57.0909091 | 17.8865831 | 55.8255488- | 58.3562693 |
|  | **t6** | 57.6363636 | 18.8689735 | 56.3015056- | 58.9712217 |
|  | **t7** | 58.6212121 | 19.4597943 | 57.2445573- | 59.9978669 |

Table A.11.2 Primary outcomes of the mixed model analyses with repeated measures: ACL-RSI (std.error= standard error, df = degrees of freedom, Diff = Difference between time points)

|  | **estimate** | **std.error** | **t value** | **df** | **p.value** | **Significance** |
| --- | --- | --- | --- | --- | --- | --- |
| (Intercept) | 35.1454 | 8.653095 | 4.061598 | 632 | <0.001 | *** |
| group | -15.4532 | 8.70684 | -1.77483 | 632 | 0.076407 |  |
| time point | 1.759194 | 1.729014 | 1.017455 | 632 | 0.309326 |  |
| ACL RSI Baseline | -0.66617 | 0.107708 | -6.18497 | 632 | <0.001 | *** |
| as.factor(time point)Diff Baseline-3 | -6.8531 | 7.67572 | -0.89283 | 632 | 0.372289 |  |
| as.factor(time point)Diff Baseline-4 | -8.23342 | 7.663656 | -1.07435 | 632 | 0.283078 |  |
| as.factor(time point)Diff Baseline-5 | -4.30965 | 7.726401 | -0.55778 | 632 | 0.57719 |  |
| as.factor(time point)Diff Baseline-6 | 0.809741 | 8.163449 | 0.099191 | 632 | 0.921018 |  |
| group: ACL RSI Baseline | 0.258904 | 0.156976 | 1.649317 | 632 | 0.09958 |  |
| group:as.factor(time point)Diff Baseline-3 | 13.93866 | 12.31333 | 1.131998 | 632 | 0.258065 |  |
| group:as.factor(time point)Diff Baseline-4 | 8.415138 | 12.57771 | 0.669052 | 632 | 0.503707 |  |
| group:as.factor(time point)Diff Baseline-5 | 6.489528 | 12.57771 | 0.515955 | 632 | 0.606066 |  |
| group:as.factor(time point)Diff Baseline-6 | -0.78565 | 12.57771 | -0.06246 | 632 | 0.950214 |  |
| group:as.factor(time point)Diff Baseline-7 | -3.08876 | 12.57771 | -0.24557 | 632 | 0.806091 |  |
| group: ACL RSI Baseline: factor(time point) Diff Baseline-3 | -0.27571 | 0.221998 | -1.24197 | 632 | 0.21471 |  |
| group: ACL RSI Baseline: factor(time point) Diff Baseline-4 | -0.08305 | 0.225636 | -0.36806 | 632 | 0.712955 |  |
| group: ACL RSI Baseline: factor(time point) Diff Baseline-5 | -0.03668 | 0.225636 | -0.16255 | 632 | 0.870923 |  |
| group: ACL RSI Baseline: factor(time point) Diff Baseline-6 | 0.037243 | 0.225636 | 0.16506 | 632 | 0.868949 |  |
| group: ACL RSI Baseline: factor(time point) Diff Baseline-7 | 0.127469 | 0.225636 | 0.564931 | 632 | 0.572321 |  |

Table A.11.3 fixed effects ACL-RSI (std.error= standard error, df = degrees of freedom)

|  | **estimate** | **std.error** | **t value** | **df** | **p.value** | **Significance** |
| --- | --- | --- | --- | --- | --- | --- |
| (Intercept) | 34.95206 | 5.249813 | 6.657773 | 581 | <0.001 | *** |
| ACL RSI Baseline | -0.58777 | 0.034875 | -16.8535 | 581 | <0.001 | *** |
| group | -3.55311 | 3.461414 | -1.02649 | 581 | 0.305088 |  |
| time point | -0.08228 | 0.514098 | -0.16004 | 581 | 0.872902 |  |
| age | 0.23922 | 0.06823 | 3.506079 | 581 | <0.001 | *** |
| time between rupture and surgery | 0.026501 | 0.01435 | 1.846751 | 581 | 0.065291 |  |
| gender | -4.36138 | 1.387419 | -3.14352 | 581 | 0.001754 | ** |
| Tegner Score before injury | -0.55529 | 0.505006 | -1.09957 | 581 | 0.271976 |  |
| group*time point | 0.848308 | 0.724675 | 1.170606 | 581 | 0.242237 |  |

# A.12 Mixed models outcomes – extension deficit

Table A.12.1 outcomes details exntension deficit (SD=standard deviation, CI=confidence interval, t1=during the initial hospital anamnesis, t2=1-7 days prior to ACL reconstruction, t3=on the day of surgery, t4= 30 days postoperatively, t5= 60 days postoperatively, t6= 90 days postoperatively and t7=180 days postoperatively)

| **group** | **time point** | **Mean** | **SD** | **CI** | |
| --- | --- | --- | --- | --- | --- |
| **comparator group** | **t1** | 4.75438596 | 7.05660962 | 4.2551764- | 5.25359553 |
|  | **t2** | 6.31578947 | 4.66046401 | 5.98609174- | 6.64548721 |
|  | **t3** | 4.28070175 | 6.02896102 | 3.85419169- | 4.70721182 |
|  | **t4** | 9.28846154 | 5.11949959 | 8.92629- | 9.65063308 |
|  | **t5** | 7.61538462 | 3.86108715 | 7.34223763- | 7.8885316 |
|  | **t6** | 7.23076923 | 5.22668637 | 6.86101492- | 7.60052354 |
|  | **t7** | 7.23076923 | 4.91741756 | 6.88289369- | 7.57864477 |
| **intervention group** | **t1** | 6.15789474 | 5.78418248 | 5.74870118- | 6.5670883 |
|  | **t2** | 4.75438596 | 3.99678756 | 4.47163905- | 5.03713288 |
|  | **t3** | 4.56140351 | 4.34255333 | 4.2541959- | 4.86861112 |
|  | **t4** | 8.2 | 4.93438427 | 7.85092418- | 8.54907582 |
|  | **t5** | 5.92727273 | 3.71111716 | 5.66473515- | 6.1898103 |
|  | **t6** | 5.94545455 | 3.6989397 | 5.68377845- | 6.20713064 |
|  | **t7** | 4.65454545 | 3.60153353 | 4.39976021- | 4.9093307 |

Table A.12.2 Primary outcomes of the mixed model analyses with repeated measures: extension deficit (std.error= standard error, df = degrees of freedom, Diff = Difference between time points)

|  | **estimate** | **std.error** | **t value** | **df** | **p value** | **Signi-ficance** |
| --- | --- | --- | --- | --- | --- | --- |
| (Intercept) | 4.280097 | 1.528124 | 2.800884 | 7.25E-09 | 1 |  |
| group | -1.71315 | 1.142308 | -1.49973 | 632 | 0.134184 |  |
| time point | 0.434391 | 0.300712 | 1.444544 | 7.64E-09 | 1 |  |
| extension deficit Baseline | -0.75456 | 0.085889 | -8.78534 | 632 | <0.001 | *** |
| as.factor(time point)Diff Baseline-3 | -1.19163 | 1.358163 | -0.87738 | 7.21E-09 | 1 |  |
| as.factor(time point)Diff Baseline-4 | 2.732389 | 1.3211 | 2.068269 | 7.88E-09 | 1 |  |
| as.factor(time point)Diff Baseline-5 | 1.022096 | 1.326234 | 0.770675 | 8.01E-09 | 1 |  |
| as.factor(time point)Diff Baseline-6 | -0.02368 | 1.397621 | -0.01694 | 8.08E-09 | 1 |  |
| group: extension deficit Baseline | -0.0313 | 0.135485 | -0.231 | 632 | 0.817387 |  |
| group:as.factor(time point)Diff Baseline-3 | 0.864207 | 1.615468 | 0.534958 | 632 | 0.592867 |  |
| group:as.factor(time point)Diff Baseline-4 | 0.135444 | 1.645833 | 0.082295 | 632 | 0.934438 |  |
| group:as.factor(time point)Diff Baseline-5 | -0.20079 | 1.645833 | -0.122 | 632 | 0.902938 |  |
| group:as.factor(time point)Diff Baseline-6 | 0.320492 | 1.645833 | 0.194729 | 632 | 0.845667 |  |
| group:as.factor(time point)Diff Baseline-7 | -1.1806 | 1.645833 | -0.71732 | 632 | 0.473439 |  |
| group: extension deficit Baseline: factor(time point) Diff Baseline-3 | 0.220063 | 0.191605 | 1.148522 | 632 | 0.251188 |  |
| group: extension deficit Baseline: factor(time point) Diff Baseline-4 | 0.091795 | 0.193125 | 0.475315 | 632 | 0.634726 |  |
| group: extension deficit Baseline: factor(time point) Diff Baseline-5 | 0.062892 | 0.193125 | 0.325654 | 632 | 0.744794 |  |
| group: extension deficit Baseline: factor(time point) Diff Baseline-6 | 0.036026 | 0.193125 | 0.186543 | 632 | 0.852079 |  |
| group: extension deficit Baseline: factor(time point) Diff Baseline-7 | 0.085591 | 0.193125 | 0.44319 | 632 | 0.65778 |  |

Table A.12.3 fixed effects extension deficit (std.error= standard error, df = degrees of freedom)

|  | **estimate** | **std.error** | **t value** | **df** | **p.value** | **Significance** |
| --- | --- | --- | --- | --- | --- | --- |
| (Intercept) | 4.811042 | 2.460648 | 1.955194 | 10.36149 | 0.078056 |  |
| extension deficit Baseline | -0.89731 | 0.031356 | -28.6163 | 577.0038 | <0.001 | *** |
| group | -0.04927 | 1.039839 | -0.04738 | 577.0091 | 0.962229 |  |
| time point | 0.341796 | 0.417139 | 0.819381 | 4.604495 | 0.452875 |  |
| age | -0.0169 | 0.020069 | -0.84234 | 576.9998 | 0.399944 |  |
| time between rupture and surgery | 0.008572 | 0.004497 | 1.906081 | 576.9993 | 0.057136 |  |
| gender | -0.56479 | 0.436951 | -1.29256 | 576.9991 | 0.196679 |  |
| Tegner Score before injury | 0.088618 | 0.150221 | 0.589922 | 577.0061 | 0.555474 |  |
| group*time point | -0.30787 | 0.217664 | -1.41443 | 577.0062 | 0.157776 |  |

# A.13 Mixed models outcomes – flexion deficit

Table A.13.1 outcomes details flexion deficit (SD=standard deviation, CI=confidence interval, t1=during the initial hospital anamnesis, t2=1-7 days prior to ACL reconstruction, t3=on the day of surgery, t4= 30 days postoperatively, t5= 60 days postoperatively, t6= 90 days postoperatively and t7=180 days postoperatively)

| **group** | **time point** | **Mean** | **SD** | **CI** | |
| --- | --- | --- | --- | --- | --- |
| **comparator group** | **t1** | 19.1929825 | 24.5265444 | 17.4578878- | 20.9280771 |
|  | **t2** | 15.0526316 | 20.0876837 | 13.6315577- | 16.4737055 |
|  | **t3** | 66.9473684 | 38.9268641 | 64.1935441- | 69.7011927 |
|  | **t4** | 29.7692308 | 22.1842687 | 28.199837- | 31.3386245 |
|  | **t5** | 19.9230769 | 22.4375789 | 18.3357631- | 21.5103907 |
|  | **t6** | 9.26923077 | 20.769619 | 7.79991434- | 10.7385472 |
|  | **t7** | 9.88461538 | 26.0833159 | 8.03938922- | 11.7298416 |
| **intervention group** | **t1** | 23.4912281 | 24.1188447 | 21.7849755- | 25.1974806 |
|  | **t2** | 14.7192982 | 19.6766308 | 13.3273037- | 16.1112928 |
|  | **t3** | 57.9649123 | 37.3463351 | 55.3229002- | 60.6069243 |
|  | **t4** | 25.7090909 | 20.6789146 | 24.2461912- | 27.1719906 |
|  | **t5** | 16.2 | 18.7630448 | 14.8726357- | 17.5273643 |
|  | **t6** | 8.14545455 | 14.5734642 | 7.11447606- | 9.17643303 |
|  | **t7** | 8.58181818 | 17.7259915 | 7.32781875- | 9.83581762 |

Table A.13.2 Primary outcomes of the mixed model analyses with repeated measures: flexion deficit (std.error= standard error, df = degrees of freedom, Diff = Difference between time points)

|  | **estimate** | **std.error** | **t value** | **df** | **p.value** | **Signi-ficance** |
| --- | --- | --- | --- | --- | --- | --- |
| (Intercept) | 12.07718 | 7.928907 | 1.523183 | <0.001 | 1 |  |
| group | 0.903432 | 6.137372 | 0.147202 | 632 | 0.88302 |  |
| time point | -0.49027 | 1.553291 | -0.31563 | <0.001 | 1 |  |
| flexion deficit Baseline | -0.79388 | 0.133263 | -5.95726 | 632 | <0.001 | *** |
| as.factor(time point)Diff Baseline-3 | 52.96435 | 7.05099 | 7.511619 | <0.001 | 1 |  |
| as.factor(time point)Diff Baseline-4 | 15.68599 | 6.805644 | 2.304851 | <0.001 | 1 |  |
| as.factor(time point)Diff Baseline-5 | 7.342621 | 6.82308 | 1.076145 | <0.001 | 1 |  |
| as.factor(time point)Diff Baseline-6 | 0.904062 | 7.18453 | 0.125835 | <0.001 | 1 |  |
| group: fleixon deficit Baseline | -0.09036 | 0.190062 | -0.47543 | 632 | 0.634643 |  |
| group:as.factor(time point)Diff Baseline-3 | -10.3333 | 8.679554 | -1.19053 | 632 | 0.234284 |  |
| group:as.factor(time point)Diff Baseline-4 | -2.96355 | 8.804697 | -0.33659 | 632 | 0.73654 |  |
| group:as.factor(time point)Diff Baseline-5 | -2.65982 | 8.804697 | -0.30209 | 632 | 0.762682 |  |
| group:as.factor(time point)Diff Baseline-6 | -4.46601 | 8.804697 | -0.50723 | 632 | 0.61217 |  |
| group:as.factor(time point)Diff Baseline-7 | -4.23348 | 8.804697 | -0.48082 | 632 | 0.63081 |  |
| group: flexion deficit Baseline: factor(time point) Diff Baseline-3 | 0.077217 | 0.268788 | 0.287278 | 632 | 0.773994 |  |
| group: flexion deficit Baseline: factor(time point) Diff Baseline-4 | -0.0362 | 0.270923 | -0.13361 | 632 | 0.893751 |  |
| group: flexion deficit Baseline: factor(time point) Diff Baseline-5 | -0.02379 | 0.270923 | -0.08782 | 632 | 0.930048 |  |
| group: flexion deficit Baseline: factor(time point) Diff Baseline-6 | 0.200437 | 0.270923 | 0.739832 | 632 | 0.459677 |  |
| group: flexion deficit Baseline: factor(time point) Diff Baseline-7 | 0.161411 | 0.270923 | 0.595781 | 632 | 0.551535 |  |

Table A.13.3 fixed effects flexion deficit (std.error= standard error, df = degrees of freedom)

|  | **estimate** | **std.error** | **t value** | **df** | **p.value** | **Significance** |
| --- | --- | --- | --- | --- | --- | --- |
| (Intercept) | 60.65565 | 25.4206 | 2.386082 | 4.796386 | 0.064848 |  |
| extension deficit Baseline | -0.88047 | 0.040152 | -21.9284 | 577.0044 | <0.001 | *** |
| group | -9.11933 | 5.167487 | -1.76475 | 577.006 | 0.078135 |  |
| time point | -5.99387 | 5.077281 | -1.18053 | 4.096848 | 0.30176 |  |
| age | -0.10576 | 0.099867 | -1.059 | 577.0045 | 0.290043 |  |
| time between rupture and surgery | -0.01265 | 0.023182 | -0.54585 | 577.0045 | 0.585379 |  |
| gender | -1.34244 | 2.071981 | -0.6479 | 577.0044 | 0.517306 |  |
| Tegner Score before injury | -0.66715 | 0.749486 | -0.89014 | 577.0054 | 0.373761 |  |
| group*time point | 0.936956 | 1.081977 | 0.865967 | 577.0055 | 0.386868 |  |

# A.14 Mixed models outcomes – Calf raise test

# Calf raise test (LSI Epic)

Table A.14.1.1 outcomes details calf raise test (LSI Epic) (SD=standard deviation, CI=confidence interval, t1=during the initial hospital anamnesis, t2=1-7 days prior to ACL reconstruction, t3=on the day of surgery, t4= 30 days postoperatively, t5= 60 days postoperatively, t6= 90 days postoperatively and t7=180 days postoperatively, LSI = limb symmetry index, EPIC = estimated pre-injury capacity)

| **group** | **time point** | **M** | **SD** | **CI** | |
| --- | --- | --- | --- | --- | --- |
| **comparator group** | **t2** | 81.42088 | 24.5095047 | 79.1112053- | 83.7305547 |
|  | **t5** | 78.8015582 | 29.9258347 | 75.9814709- | 81.6216455 |
|  | **t6** | 87.2096531 | 23.6500012 | 84.9809745- | 89.4383317 |
|  | **t7** | 90.5489887 | 30.0624901 | 87.7160235- | 93.3819538 |
| **intervention group** | **t2** | 86.8241758 | 21.646744 | 84.7842759- | 88.8640757 |
|  | **t5** | 82.5529354 | 27.1831656 | 79.9913059- | 85.1145648 |
|  | **t6** | 84.541321 | 24.6351524 | 82.2198058- | 86.8628362 |
|  | **t7** | 92.4866165 | 20.3406047 | 90.5698018- | 94.4034312 |

Table A.14.1.2 Primary outcomes of the mixed model analyses with repeated measures: calf raise test (LSI Epic) (std.error= standard error, df = degrees of freedom, Diff = Difference between time points)

|  | **estimate** | **std.error** | **t value** | **df** | **p.value** | **Signif-icance** |
| --- | --- | --- | --- | --- | --- | --- |
| (Intercept) | -69.9844 | 53.65333 | -1.30438 | <0.001 | 0.999997 |  |
| group | 5.900863 | 18.53413 | 0.318378 | 308.9995 | 0.750413 |  |
| time point | 25.77729 | 8.820552 | 2.922412 | <0.001 | 0.999997 |  |
| calf raise test LSI Epic Baseline | -0.753 | 0.142086 | -5.29957 | 308.998 | <0.001 | *** |
| as.factor(time point)Diff Baseline-6 | 3.912999 | 15.27764 | 0.256126 | <0.001 | 0.999998 |  |
| group: calf raise test LSI Epic Baseline | -0.04143 | 0.213102 | -0.19442 | 308.9996 | 0.845972 |  |
| group:as.factor(time point)Diff Baseline-6 | -43.4857 | 26.21122 | -1.65905 | 308.9999 | 0.098121 |  |
| group:as.factor(time point)Diff Baseline-7 | -60.4025 | 26.21122 | -2.30445 | 308.9994 | 0.02186 | * |
| group: calf raise test LSI Epic Baseline: factor(time point) Diff Baseline-6 | 0.446964 | 0.301372 | 1.483099 | 309 | 0.139067 |  |
| group: calf raise test LSI Epic Baseline: factor(time point) Diff Baseline-7 | 0.711624 | 0.301372 | 2.361285 | 308.9994 | 0.018832 | * |

Table A.14.1.3 fixed effects calf raise test (LSI Epic) (std.error= standard error, df = degrees of freedom)

|  | **estimate** | **std.error** | **t value** | **df** | **p.value** | **Significance** |
| --- | --- | --- | --- | --- | --- | --- |
| (Intercept) | 50.84588 | 21.03156 | 2.417599 | 279 | 0.016264 | * |
| calf raise test LSI Epic Baseline | -0.87081 | 0.065585 | -13.2776 | 279 | <0.001 | *** |
| group | -0.34626 | 22.8225 | -0.01517 | 279 | 0.987906 |  |
| time point | 6.456941 | 2.692087 | 2.398489 | 279 | 0.01712 | * |
| age | -0.30647 | 0.168439 | -1.8195 | 279 | 0.069907 |  |
| time between rupture and surgery | -0.02621 | 0.03516 | -0.74546 | 279 | 0.456622 |  |
| gender | -3.6757 | 3.425611 | -1.07301 | 279 | 0.284196 |  |
| Tegner Score before injury | 0.273389 | 1.236925 | 0.221023 | 279 | 0.825236 |  |
| group*time point | 0.299478 | 3.768137 | 0.079476 | 279 | 0.936711 |  |

# Calf raise test (LSI)

Table A.14.2.1 outcomes details calf raise test (LSI) (SD=standard deviation, CI=confidence interval, t1=during the initial hospital anamnesis, t2=1-7 days prior to ACL reconstruction, t3=on the day of surgery, t4= 30 days postoperatively, t5= 60 days postoperatively, t6= 90 days postoperatively and t7=180 days postoperatively, LSI = limb symmetry index)

| **group** | **time point** | **Mean** | **SD** | **CI** | |
| --- | --- | --- | --- | --- | --- |
| **comparator group** | **t2** | 82.1031412 | 25.0292875 | 79.7444843- | 84.4617981 |
|  | **t5** | 80.1132684 | 29.0737656 | 77.3734766- | 82.8530602 |
|  | **t6** | 86.1334041 | 22.6437349 | 83.9995519- | 88.2672564 |
|  | **t7** | 91.6035091 | 29.6778384 | 88.806792- | 94.4002263 |
| **intervention group** | **t2** | 87.635313 | 22.0839758 | 85.5542101- | 89.7164158 |
|  | **t5** | 83.5843656 | 26.3880009 | 81.0976692- | 86.071062 |
|  | **t6** | 84.8232474 | 23.2511418 | 82.6321556- | 87.0143391 |
|  | **t7** | 94.6560651 | 19.7027364 | 92.7993605- | 96.5127698 |


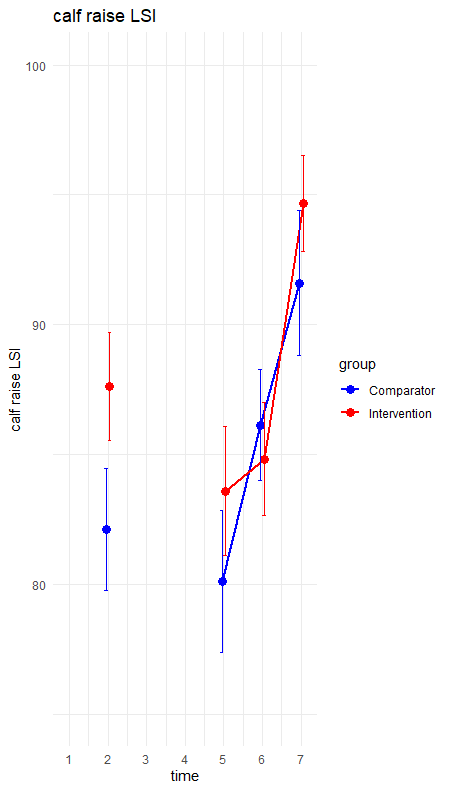


Figure A.14.2 Means and 95% confidence intervals (CI) of the calf raise test (LSI), separated by groups. The x-axis shows the 7 measurement time points, (t1=during the initial hospital anamnesis, t2=1-7 days prior to ACL reconstruction, t3=on the day of surgery, t4= 30 days postoperatively, t5= 60 days postoperatively, t6= 90 days postoperatively and t7=180 days postoperatively), the y-axis shows the values of the LSI and 95% CI; (LSI = limb symmetry index)

Table A.14.2.2 Primary outcomes of the mixed model analyses with repeated measures: calf raise test (LSI) (std.error= standard error, df = degrees of freedom, Diff = Difference between time points)

|  | **estimate** | **std.error** | **t value** | **df** | **p.value** | **Signi-ficance** |
| --- | --- | --- | --- | --- | --- | --- |
| (Intercept) | -44.2626 | 57.13498 | -0.7747 | 7.77E-08 | 0.999999 |  |
| group | 18.2493 | 18.07024 | 1.009909 | 308.9996 | 0.313329 |  |
| time point | 20.53602 | 9.392933 | 2.186326 | 7.77E-08 | 0.999999 |  |
| calf raise test LSI Baseline | -0.73318 | 0.136962 | -5.35316 | 308.9981 | <0.001 | *** |
| as.factor(time point)Diff Baseline-6 | 1.93472 | 16.26904 | 0.11892 | 7.77E-08 | 0.999999 |  |
| group: calf raise test LSI Baseline | -0.18748 | 0.20561 | -0.91182 | 308.9996 | 0.362574 |  |
| group:as.factor(time point)Diff Baseline-6 | -33.2156 | 25.55518 | -1.29976 | 308.9999 | 0.194652 |  |
| group:as.factor(time point)Diff Baseline-7 | -43.4192 | 25.55518 | -1.69904 | 308.9986 | 0.090319 |  |
| group: calf raise test LSI Baseline: factor(time point) Diff Baseline-6 | 0.339766 | 0.290776 | 1.16848 | 308.9999 | 0.243514 |  |
| group: calf raise test LSI Baseline: factor(time point) Diff Baseline-7 | 0.517718 | 0.290776 | 1.780471 | 308.9987 | 0.075981 |  |

Table A.14.2.3 fixed effects calf raise test (LSI) (std.error= standard error, df = degrees of freedom)

|  | **estimate** | **std.error** | **t value** | **df** | **p.value** | **Significance** |
| --- | --- | --- | --- | --- | --- | --- |
| (Intercept) | 65.11773 | 20.16531 | 3.229196 | 279 | 0.00139 | ** |
| calf raise test LSI Baseline | -0.9021 | 0.061305 | -14.7149 | 279 | <0.001 | *** |
| group | -12.2048 | 21.96217 | -0.55572 | 279 | 0.57885 |  |
| time point | 5.278622 | 2.590627 | 2.037585 | 279 | 0.042535 | * |
| age | -0.30492 | 0.160939 | -1.89462 | 279 | 0.059177 |  |
| time between rupture and surgery | -0.00209 | 0.033848 | -0.06164 | 279 | 0.950897 |  |
| gender | -3.93921 | 3.297048 | -1.19477 | 279 | 0.233192 |  |
| Tegner Score before injury | -0.58444 | 1.189563 | -0.49131 | 279 | 0.623596 |  |
| group*time point | 2.410915 | 3.626122 | 0.664874 | 279 | 0.50668 |  |

# A.15 Mixed models outcomes – Y-Balance Test

# Y-Balance LSI Epic

Table A.15.1.1 outcomes details Y-Balance LSI Epic (SD=standard deviation, CI=confidence interval, t1=during the initial hospital anamnesis, t2=1-7 days prior to ACL reconstruction, t3=on the day of surgery, t4= 30 days postoperatively, t5= 60 days postoperatively, t6= 90 days postoperatively and t7=180 days postoperatively, LSI = limb symmetry index, EPIC = estimated pre-injury capacity)

| **group** | **time point** | **Mean** | **SD** | **CI** | |
| --- | --- | --- | --- | --- | --- |
| **comparator group** | **t2** | 97.2598944 | 6.80180237 | 96.6189206- | 97.9008682 |
|  | **t5** | 97.6317943 | 9.80418762 | 96.7078881- | 98.5557005 |
|  | **t6** | 98.6476691 | 7.50746391 | 97.9401966- | 99.3551415 |
|  | **t7** | 99.0409752 | 7.78119186 | 98.3077078- | 99.7742427 |
| **intervention group** | **t2** | 97.2288931 | 8.29530333 | 96.4471779- | 98.0106083 |
|  | **t5** | 97.075098 | 8.94836946 | 96.2318405- | 97.9183554 |
|  | **t6** | 99.9832625 | 14.1991334 | 98.6451947- | 101.32133 |
|  | **t7** | 102.43252 | 9.15127987 | 101.570141- | 103.294899 |

Table A.15.1.2 Primary outcomes of the mixed model analyses with repeated measures: Y-Balance Test (LSI Epic) (std.error= standard error, df = degrees of freedom, Diff = Difference between time points)

|  | **estimate** | **std.error** | **t value** | **df** | **p.value** | **Signifi-cance** |
| --- | --- | --- | --- | --- | --- | --- |
| (Intercept) | 202.5242 | 89.83701 | 2.254352 | 0.135189 | 0.716799 |  |
| group | -26.9331 | 25.98088 | -1.03665 | 308.9816 | 0.30071 |  |
| time point | -15.7964 | 14.76911 | -1.06956 | 0.135187 | 0.790352 |  |
| Y Balance test LSI Epic Baseline | -1.2667 | 0.214479 | -5.90594 | 308.9555 | <0.001 | *** |
| as.factor(time point)Diff Baseline-6 | -19.1447 | 25.58085 | -0.7484 | 0.135079 | 0.826296 |  |
| group: Y Balance test LSI Epic Baseline | 0.271497 | 0.266683 | 1.018051 | 308.9817 | 0.30945 |  |
| group:as.factor(time point)Diff Baseline-6 | 73.54708 | 36.74252 | 2.001689 | 308.9944 | 0.046192 | * |
| group:as.factor(time point)Diff Baseline-7 | 26.7924 | 36.74252 | 0.729193 | 308.9506 | 0.466436 |  |
| group: Y Balance test LSI Epic Baseline: factor(time point) Diff Baseline-6 | -0.73745 | 0.377146 | -1.95535 | 308.9945 | 0.051443 |  |
| group: Y Balance test LSI Epic Baseline: factor(time point) Diff Baseline-7 | -0.23517 | 0.377146 | -0.62355 | 308.9507 | 0.533382 |  |

Table A.15.1.3 fixed effects Y-Balance Test (LSI Epic) (std.error= standard error, df = degrees of freedom)

|  | **estimate** | **std.error** | **t value** | **df** | **p.value** | **Significance** |
| --- | --- | --- | --- | --- | --- | --- |
| (Intercept) | 91.13934 | 11.47609 | 7.941674 | 279 | <0.001 | *** |
| Y Balance test LSI Epic Baseline | -0.99097 | 0.085917 | -11.534 | 279 | <0.001 | *** |
| group | -9.57561 | 8.749784 | -1.09438 | 279 | 0.274731 |  |
| time point | 0.758906 | 1.032239 | 0.735204 | 279 | 0.462833 |  |
| age | 0.114452 | 0.064073 | 1.78628 | 279 | 0.07514 |  |
| time between rupture and surgery | -0.00834 | 0.013493 | -0.61839 | 279 | 0.536822 |  |
| gender | -1.09435 | 1.314329 | -0.83263 | 279 | 0.405767 |  |
| Tegner Score before injury | 0.110411 | 0.473091 | 0.233381 | 279 | 0.815636 |  |
| group*time point | 1.803409 | 1.444833 | 1.248178 | 279 | 0.213012 |  |

# Y-Balance LSI

Table A.15.2.1 outcomes details Y-Balance LSI (SD=standard deviation, CI=confidence interval, t1=during the initial hospital anamnesis, t2=1-7 days prior to ACL reconstruction, t3=on the day of surgery, t4= 30 days postoperatively, t5= 60 days postoperatively, t6= 90 days postoperatively and t7=180 days postoperatively, LSI = limb symmetry index)

| **group** | **time point** | **Mean** | **SD** | **CI** | |
| --- | --- | --- | --- | --- | --- |
| **comparator group** | **t2** | 98.3268842 | 5.76081374 | 97.7840088- | 98.8697595 |
|  | **t5** | 96.3635149 | 6.43109141 | 95.7574754- | 96.9695544 |
|  | **t6** | 98.5725894 | 5.64967515 | 98.0401873- | 99.1049915 |
|  | **t7** | 96.9149674 | 5.22434603 | 96.4226466- | 97.4072883 |
| **intervention group** | **t2** | 98.4115883 | 6.6184635 | 97.7878916- | 99.035285 |
|  | **t5** | 96.9374416 | 6.69559999 | 96.3064758- | 97.5684073 |
|  | **t6** | 97.4465763 | 7.02001349 | 96.7850392- | 98.1081135 |
|  | **t7** | 99.107511 | 6.07269497 | 98.5352452- | 99.6797767 |


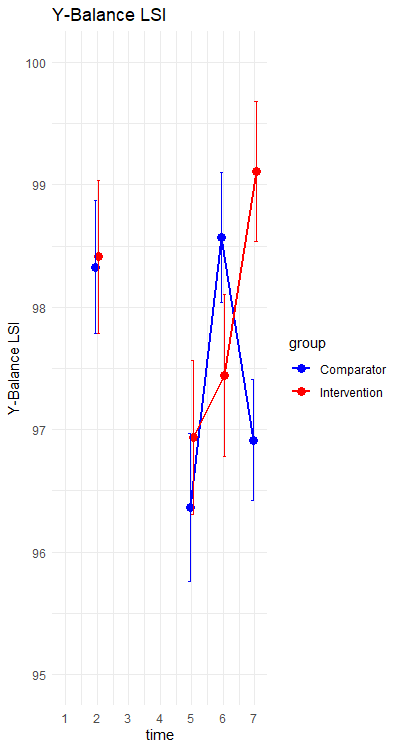


Figure A.15.2 Means and 95% confidence intervals (CI) of the Y Balance test (LSI), separated by groups. The x-axis shows the 7 measurement time points, (t1=during the initial hospital anamnesis, t2=1-7 days prior to ACL reconstruction, t3=on the day of surgery, t4= 30 days postoperatively, t5= 60 days postoperatively, t6= 90 days postoperatively and t7=180 days postoperatively), the y-axis shows the values of the LSI and 95% CI; (LSI = limb symmetry index).

Table A.15.2.2 Primary outcomes of the mixed model analyses with repeated measures: Y-Balance Test (LSI) (std.error= standard error, df = degrees of freedom, Diff = Difference between time points)

|  | **estimate** | **std.error** | **t value** | **df** | **p.value** | **Signifi-cance** |
| --- | --- | --- | --- | --- | --- | --- |
| (Intercept) | 113.694 | 67.68274 | 1.679807 | 5.776684 | 0.145909 |  |
| group | -13.8713 | 19.69193 | -0.70441 | 308.9997 | 0.481706 |  |
| time point | -5.09824 | 11.12697 | -0.45819 | 5.775026 | 0.663551 |  |
| Y Balance test LSI Baseline | -0.917 | 0.159823 | -5.73761 | 308.9985 | <0.001 | *** |
| as.factor(time point)Diff Baseline-6 | 0.438053 | 19.27248 | 0.022729 | 5.775141 | 0.982631 |  |
| group: Y Balance test LSI Baseline | 0.146933 | 0.199947 | 0.734862 | 308.9997 | 0.462981 |  |
| group:as.factor(time point)Diff Baseline-6 | -31.7573 | 27.84859 | -1.14036 | 308.999 | 0.255021 |  |
| group:as.factor(time point)Diff Baseline-7 | 4.619642 | 27.84859 | 0.165884 | 308.9998 | 0.868357 |  |
| group: Y Balance test LSI Baseline: factor(time point) Diff Baseline-6 | 0.305731 | 0.282768 | 1.081207 | 308.999 | 0.280448 |  |
| group: Y Balance test LSI Baseline: factor(time point) Diff Baseline-7 | -0.03052 | 0.282768 | -0.10793 | 308.9998 | 0.914125 |  |

Table A.15.2.3 fixed effects Y-Balance Test (LSI) (std.error= standard error, df = degrees of freedom)

|  | **estimate** | **std.error** | **t value** | **df** | **p.value** | **Significance** |
| --- | --- | --- | --- | --- | --- | --- |
| (Intercept) | 71.94606 | 8.230377 | 8.741527 | 279 | <0.001 | *** |
| Y Balance test LSI Baseline | -0.69855 | 0.063594 | -10.9846 | 279 | <0.001 | *** |
| group | -5.17639 | 5.287912 | -0.97891 | 279 | 0.328473 |  |
| time point | 0.2382 | 0.623822 | 0.38184 | 279 | 0.702871 |  |
| age | -0.034 | 0.040492 | -0.83974 | 279 | 0.401772 |  |
| time between rupture and surgery | -0.00234 | 0.008156 | -0.28666 | 279 | 0.774583 |  |
| gender | -1.43232 | 0.793883 | -1.80419 | 279 | 0.07228 |  |
| Tegner Score before injury | -0.32814 | 0.287924 | -1.13966 | 279 | 0.255404 |  |
| group*time point | 0.937195 | 0.873168 | 1.073327 | 279 | 0.284052 |  |

# A.16 Mixed models outcomes – isokinetic testing

# HQ-Ratio – Peak Torque (60°)

Table A.16.1.1 outcomes details HQ-Ratio Peak Torque (60°), (SD=standard deviation, CI=confidence interval, t1=during the initial hospital anamnesis, t2=1-7 days prior to ACL reconstruction, t3=on the day of surgery, t4= 30 days postoperatively, t5= 60 days postoperatively, t6= 90 days postoperatively and t7=180 days postoperatively, HQ-Ratio = Hamstrings-Quadriceps-Ratio)

| **group** | **time point** | **Mean** | **SD** | **CI** | |
| --- | --- | --- | --- | --- | --- |
| **comparator group** | **t6** | 0.82917735 | 0.35442315 | 0.76091969- | 0.89743502 |
|  | **t7** | 0.78980143 | 0.24687381 | 0.74225648- | 0.83734637 |
| **intervention group** | **t6** | 0.89595708 | 0.24267302 | 0.84922116- | 0.942693 |
|  | **t7** | 0.76810977 | 0.17334559 | 0.73472548- | 0.80149406 |

Table A.16.1.2 Primary outcomes of the mixed model analyses with repeated measures: HQ-Ratio Peak-Toruqe (60°) (std.error= standard error, df = degrees of freedom, Diff = Difference between time points)

|  | **estimate** | **std.error** | **t value** | **df** | **p.value** | **Significance** |
| --- | --- | --- | --- | --- | --- | --- |
| (Intercept) | 1.065433 | 1.223928 | 0.870503 | <0.001 | 1 |  |
| group | 0.06678 | 0.069811 | 0.956577 | 102 | 0.341042 |  |
| time point | -0.03938 | 0.187946 | -0.20951 | <0.001 | 1 |  |
| group:as.factor(time point)Diff Baseline-7 | -0.08847 | 0.101517 | -0.87149 | 102 | 0.385532 |  |

Table A.16.1.3 fixed effects Y-Balance Test: HQ-Ratio Peak-Toruqe (60°) (std.error= standard error, df = degrees of freedom)

|  | **estimate** | **std.error** | **t value** | **df** | **p.value** | **Significance** |
| --- | --- | --- | --- | --- | --- | --- |
| (Intercept) | 1.038955 | 0.707293 | 1.468917 | <0.001 | 1 |  |
| group | 0.654738 | 0.654553 | 1.000283 | 97.99989 | 0.319637 |  |
| time point | -0.0369 | 0.104716 | -0.35237 | <0.001 | 1 |  |
| age | 0.00372 | 0.002675 | 1.390727 | 98 | 0.167458 |  |
| time between rupture and surgery | -0.00092 | 0.000584 | -1.57892 | 98 | 0.117576 |  |
| gender | 0.083872 | 0.052132 | 1.608858 | 98 | 0.110864 |  |
| Tegner Score before injury | -0.02134 | 0.0202 | -1.05636 | 98 | 0.293399 |  |
| group*time point | -0.10017 | 0.100949 | -0.99228 | 97.99989 | 0.323507 |  |

# HQ-Ratio – sum of torques (180°)

Table A.16.2.1 outcomes details HQ-Ratio – sum of torques (180°), (SD=standard deviation, CI=confidence interval, t1=during the initial hospital anamnesis, t2=1-7 days prior to ACL reconstruction, t3=on the day of surgery, t4= 30 days postoperatively, t5= 60 days postoperatively, t6= 90 days postoperatively and t7=180 days postoperatively, HQ-Ratio = Hamstrings-Quadricpes Ratio)

| **group** | **time point** | **Mean** | **SD** | **CI** | |
| --- | --- | --- | --- | --- | --- |
| **comparator group** | **t6** | 1.15250769 | 0.3891526 | 1.07756155 | 1.22745383 |
|  | **t7** | 1.0840613 | 0.38656915 | 1.0096127 | 1.15850989 |
| **intervention group** | **t6** | 1.14252126 | 0.43150009 | 1.0594195 | 1.22562302 |
|  | **t7** | 0.93637715 | 0.26655849 | 0.88504118 | 0.98771313 |


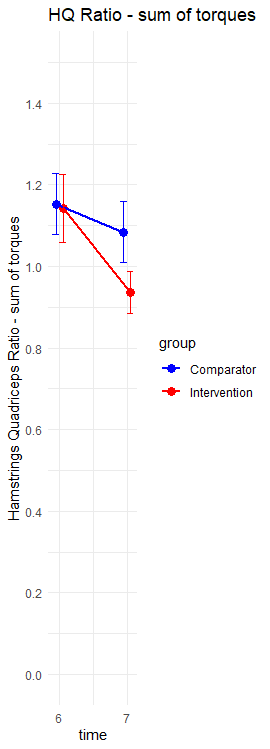


Figure A.16.2 Means and 95% confidence intervals (CI) of the isokinetic testing Hamstrings-Quadriceps Ratio (HQ-Ratio) (sum of torques), separated by groups. The x-axis shows the 7 measurement time points, (t1=during the initial hospital anamnesis, t2=1-7 days prior to ACL reconstruction, t3=on the day of surgery, t4= 30 days postoperatively, t5= 60 days postoperatively, t6= 90 days postoperatively and t7=180 days postoperatively), the y-axis shows the values of the HQ-Ratio and 95% CI.

Table A.16.2.2 Primary outcomes of the mixed model analyses with repeated measures: sum of torques (180°) (std.error= standard error, df = degrees of freedom, Diff = Difference between time points)

|  | **estimate** | **std.error** | **t value** | **df** | **p.value** | **Significance** |
| --- | --- | --- | --- | --- | --- | --- |
| (Intercept) | 1.563186 | 1.49881 | 1.042952 | <0.001 | 1 |  |
| group | -0.00999 | 0.102778 | -0.09717 | 102 | 0.922786 |  |
| time point | -0.06845 | 0.230269 | -0.29725 | <0.001 | 1 |  |
| group:as.factor(time point)Diff Baseline-7 | -0.1377 | 0.149456 | -0.92133 | 102 | 0.359053 |  |

Table A.16.2.3 fixed effects Y-Balance Test: sum of torques (180°) (std.error= standard error, df = degrees of freedom)

|  | **estimate** | **std.error** | **t value** | **df** | **p.value** | **Significance** |
| --- | --- | --- | --- | --- | --- | --- |
| (Intercept) | 1.805876 | 1.454747 | 1.241367 | <0.001 | 1 |  |
| group | 0.996644 | 0.928346 | 1.073569 | 97.99988 | 0.285652 |  |
| time point | -0.06081 | 0.219487 | -0.27704 | <0.001 | 1 |  |
| age | 0.006253 | 0.003794 | 1.648126 | 98 | 0.102529 |  |
| time between rupture and surgery | -0.00211 | 0.000829 | -2.54148 | 98 | 0.012606 | * |
| gender | 0.106177 | 0.073938 | 1.436029 | 98 | 0.154178 |  |
| Tegner Score before injury | -0.06653 | 0.02865 | -2.32206 | 98 | 0.0223 | * |
| group*time point | -0.17143 | 0.143175 | -1.19736 | 97.99988 | 0.234055 |  |

# A.17 Mixed models outcomes – CMJ

# CMJ- height

Table A.17.1.1 outcomes details CMJ height in cm (SD=standard deviation, CI=confidence interval, t1=during the initial hospital anamnesis, t2=1-7 days prior to ACL reconstruction, t3=on the day of surgery, t4= 30 days postoperatively, t5= 60 days postoperatively, t6= 90 days postoperatively and t7=180 days postoperatively, CMJ = counter movement jump)

| **group** | **time point** | **M** | **SD** | **CI** | |
| --- | --- | --- | --- | --- | --- |
| **comparator group** | **t6** | 0.2352381 | 0.06645442 | 0.22231437 | 0.24816182 |
|  | **t7** | 0.252 | 0.08237271 | 0.23598056 | 0.26801944 |
| **intervention group** | **t6** | 0.22 | 0.07416198 | 0.20557734 | 0.23442266 |
|  | **t7** | 0.255625 | 0.08397148 | 0.23929464 | 0.27195536 |


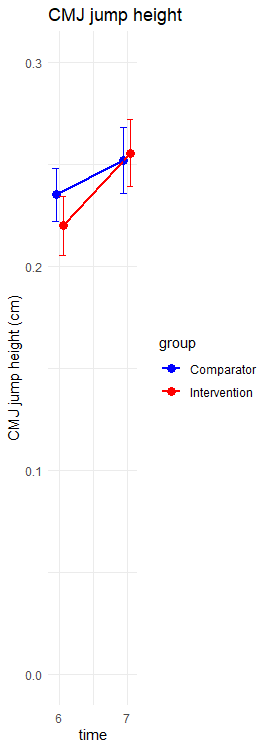


Figure A.17.1 Means and 95% confidence intervals (CI) of the Counter Movement Jump (CMJ) – jump height in cm, separated by groups. The x-axis shows the 7 measurement time points, (t1=during the initial hospital anamnesis, t2=1-7 days prior to ACL reconstruction, t3=on the day of surgery, t4= 30 days postoperatively, t5= 60 days postoperatively, t6= 90 days postoperatively and t7=180 days postoperatively), the y-axis shows the values of the jump height in cm and 95% CI.

Table A.17.1.2 Primary outcomes of the mixed model analyses with repeated measures: CMJ height (in cm) (std.error= standard error, df = degrees of freedom, Diff = Difference between time points)

|  | **estimate** | **std.error** | **t value** | **df** | **p.value** | **Significance** |
| --- | --- | --- | --- | --- | --- | --- |
| (Intercept) | 0.134667 | 0.195205 | 0.689872 | <0.001 | 1 |  |
| group | -0.01524 | 0.021907 | -0.69557 | 100 | 0.488309 |  |
| time point | 0.016762 | 0.02998 | 0.55911 | <0.001 | 1 |  |
| group:as.factor(time point)Diff Baseline-7 | 0.018863 | 0.031114 | 0.606251 | 100 | 0.545722 |  |

Table A.17.1.3 fixed effects Y-Balance Test: CMJ height (in cm) (std.error= standard error, df = degrees of freedom)

|  | **estimate** | **std.error** | **t value** | **df** | **p.value** | **Significance** |
| --- | --- | --- | --- | --- | --- | --- |
| (Intercept) | 0.052595 | 0.120237 | 0.437425 | 96 | 0.662786 |  |
| group | -0.12825 | 0.142324 | -0.90114 | 96 | 0.36977 |  |
| time point | 0.021389 | 0.017068 | 1.253143 | 96 | 0.213197 |  |
| age | -0.00244 | 0.000599 | -4.08121 | 96 | <0.001 | *** |
| time between rupture and surgery | <0.001 | 0.000133 | -0.22057 | 96 | 0.825896 |  |
| gender | 0.094734 | 0.011772 | 8.047261 | 96 | <0.001 | *** |
| Tegner Score before injury | -0.00232 | 0.004543 | -0.51115 | 96 | 0.610419 |  |
| group*time point | 0.018691 | 0.021863 | 0.854917 | 96 | 0.394726 |  |

# CMJ – LSI

Table A.17.2.1 outcomes details CMJ – LSI, (SD=standard deviation, CI=confidence interval, t1=during the initial hospital anamnesis, t2=1-7 days prior to ACL reconstruction, t3=on the day of surgery, t4= 30 days postoperatively, t5= 60 days postoperatively, t6= 90 days postoperatively and t7=180 days postoperatively, CMJ = counter movement jump, LSI = limb symmetry index)

| **group** | **time point** | **Mean** | **SD** | **CI** | |
| --- | --- | --- | --- | --- | --- |
| **comparator group** | **t6** | 89.9721464 | 32.8548121 | 83.5510152 | 96.3932776 |
|  | **t7** | 91.6017487 | 21.4687456 | 87.4059057 | 95.7975916 |
| **intervention group** | **t6** | 85.0752317 | 19.6964969 | 81.2257564 | 88.9247071 |
|  | **t7** | 90.7624005 | 13.3160353 | 88.1599199 | 93.364881 |

Table A.17.2.2 Primary outcomes of the mixed model analyses with repeated measures: CMJ LSI (std.error= standard error, df = degrees of freedom, Diff = Difference between time points)

|  | **estimate** | **std.error** | **t value** | **df** | **p.value** | **Significance** |
| --- | --- | --- | --- | --- | --- | --- |
| (Intercept) | 80.19453 | 55.12722 | 1.454718 | 99 | 0.148911 |  |
| group | -4.89691 | 6.205509 | -0.78912 | 99 | 0.431925 |  |
| time point | 1.629602 | 8.466365 | 0.19248 | 99 | 0.847761 |  |
| group:as.factor(time point)Diff Baseline-7 | 4.057566 | 8.783977 | 0.461928 | 99 | 0.645146 |  |

Table A.17.2.3 fixed effects Y-Balance Test: CMJ LSI (std.error= standard error, df = degrees of freedom)

|  | **estimate** | **std.error** | **t value** | **df** | **p.value** | **Significance** |
| --- | --- | --- | --- | --- | --- | --- |
| (Intercept) | 81.95762 | 52.24974 | 1.568575 | <0.001 | 1 |  |
| group | -32.6209 | 57.86258 | -0.56377 | 94.99999 | 0.574242 |  |
| time point | 1.000253 | 7.496143 | 0.133436 | <0.001 | 1 |  |
| age | -0.03507 | 0.243146 | -0.14423 | 95 | 0.88562 |  |
| time between rupture and surgery | 0.017207 | 0.053998 | 0.31867 | 95 | 0.750676 |  |
| gender | 6.74863 | 4.796774 | 1.40691 | 95 | 0.162717 |  |
| Tegner Score before injury | -1.24614 | 1.848279 | -0.67421 | 95 | 0.501812 |  |
| group*time point | 4.573383 | 8.884246 | 0.514774 | 94.99999 | 0.607906 |  |

# A.18 Consort Checklist


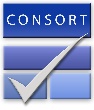
CONSORT 2010 checklist of information to include when reporting a randomised trial*

| Section/Topic | Item No | Checklist item | Reported on page No |
| --- | --- | --- | --- |
| Title and abstract | | | |
|  | 1a | Identification as a randomised trial in the title | Page 01 |
|  | 1b | Structured summary of trial design, methods, results, and conclusions (for specific guidance see CONSORT for abstracts) | Page 03 |
| Introduction | | | |
| Background and objectives | 2a | Scientific background and explanation of rationale | Page 05-06 |
|  | 2b | Specific objectives or hypotheses | Page 06 |
| Methods | | | |
| Trial design | 3a | Description of trial design (such as parallel, factorial) including allocation ratio | Page 06 |
|  | 3b | Important changes to methods after trial commencement (such as eligibility criteria), with reasons | Page 06 |
| Participants | 4a | Eligibility criteria for participants | Page 07 |
|  | 4b | Settings and locations where the data were collected | Page 07-08 |
| Interventions | 5 | The interventions for each group with sufficient details to allow replication, including how and when they were actually administered | Page 08-10 |
| Outcomes | 6a | Completely defined pre-specified primary and secondary outcome measures, including how and when they were assessed | Page 10-12 |
|  | 6b | Any changes to trial outcomes after the trial commenced, with reasons | Page 06 |
| Sample size | 7a | How sample size was determined | Page 06 |
|  | 7b | When applicable, explanation of any interim analyses and stopping guidelines | Not applicable |
| Randomisation: |  |  |  |
| Sequence generation | 8a | Method used to generate the random allocation sequence | Page 06 |
|  | 8b | Type of randomisation; details of any restriction (such as blocking and block size) | Page 06 |
| Allocation concealment mechanism | 9 | Mechanism used to implement the random allocation sequence (such as sequentially numbered containers), describing any steps taken to conceal the sequence until interventions were assigned | Page 06 |
| Implementation | 10 | Who generated the random allocation sequence, who enrolled participants, and who assigned participants to interventions | Page 06-07 |
| Blinding | 11a | If done, who was blinded after assignment to interventions (for example, participants, care providers, those assessing outcomes) and how | Page 06 |
|  | 11b | If relevant, description of the similarity of interventions | Page 08-10 |
| Statistical methods | 12a | Statistical methods used to compare groups for primary and secondary outcomes | Page 12 |
|  | 12b | Methods for additional analyses, such as subgroup analyses and adjusted analyses | Page 12 |
| Results | | | |
| Participant flow (a diagram is strongly recommended) | 13a | For each group, the numbers of participants who were randomly assigned, received intended treatment, and were analysed for the primary outcome | Page 13 |
|  | 13b | For each group, losses and exclusions after randomisation, together with reasons | Page 13, figure |
| Recruitment | 14a | Dates defining the periods of recruitment and follow-up | Page 13, figure |
|  | 14b | Why the trial ended or was stopped | Page 6, 13, figures |
| Baseline data | 15 | A table showing baseline demographic and clinical characteristics for each group | Page 13-15 |
| Numbers analysed | 16 | For each group, number of participants (denominator) included in each analysis and whether the analysis was by original assigned groups | Page 15-17, figures |
| Outcomes and estimation | 17a | For each primary and secondary outcome, results for each group, and the estimated effect size and its precision (such as 95% confidence interval) | Page 15-18, figures |
|  | 17b | For binary outcomes, presentation of both absolute and relative effect sizes is recommended | Page 15-18, figures |
| Ancillary analyses | 18 | Results of any other analyses performed, including subgroup analyses and adjusted analyses, distinguishing pre-specified from exploratory | Supplement |
| Harms | 19 | All important harms or unintended effects in each group (for specific guidance see CONSORT for harms) | Page 13 |
| Discussion | | | |
| Limitations | 20 | Trial limitations, addressing sources of potential bias, imprecision, and, if relevant, multiplicity of analyses | Page 18-24 |
| Generalisability | 21 | Generalisability (external validity, applicability) of the trial findings | Page 18-19 |
| Interpretation | 22 | Interpretation consistent with results, balancing benefits and harms, and considering other relevant evidence | Page 18-24 |
| Other information | | |  |
| Registration | 23 | Registration number and name of trial registry | Page 03-04 |
| Protocol | 24 | Where the full trial protocol can be accessed, if available | Page 06 |
| Funding | 25 | Sources of funding and other support (such as supply of drugs), role of funders | Page 04 |

Citation: Schulz KF, Altman DG, Moher D, for the CONSORT Group. CONSORT 2010 Statement: updated guidelines for reporting parallel group randomised trials. BMC Medicine. 2010;8:18.

© 2010 Schulz et al. This is an Open Access article distributed under the terms of the Creative Commons Attribution License (http://creativecommons.org/licenses/by/2.0), which permits unrestricted use, distribution, and reproduction in any medium, provided the original work is properly cited.

# A.19 Checklist TIDier (Template for Intervention Description and Replication)

**
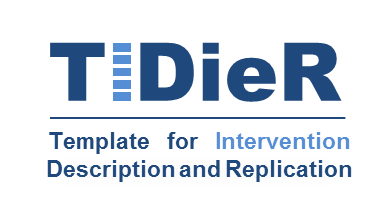
The TIDieR (Template for Intervention Description and Replication) Checklist*:**

Information to include when describing an interventionand the location of the information

| **Item number** | **Item** | **Where located **** | |
| --- | --- | --- | --- |
|  |  | Primary  paper  (page or  appendix  number) | Other ^†^ (details): Study protocol: **doi:10.1186/s13063-023-07776-1** |
|  | **BRIEF NAME** |  |  |
| **1.** | Provide the name or a phrase that describes the intervention. | Page 08 |  |
|  | **WHY** |  |  |
| **2.** | Describe any rationale, theory, or goal of the elements essential to the intervention. | Page 08-09 |  |
|  | **WHAT** |  |  |
| **3.** | Materials: Describe any physical or informational materials used in the intervention, including those provided to participants or used in intervention delivery or in training of intervention providers. Provide information on where the materials can be accessed (e.g. online appendix, URL). |  | See study protocol. |
| **4.** | Procedures: Describe each of the procedures, activities, and/or processes used in the intervention, including any enabling or support activities. | Page 08-10 | Details, see study protocol. |
|  | **WHO PROVIDED** |  |  |
| **5.** | For each category of intervention provider (e.g. psychologist, nursing assistant), describe their expertise, background and any specific training given. |  | See study protocol. |
|  | **HOW** |  |  |
| **6.** | Describe the modes of delivery (e.g. face-to-face or by some other mechanism, such as internet or telephone) of the intervention and whether it was provided individually or in a group. | Page 08-10 |  |
|  | **WHERE** |  |  |
| **7.** | Describe the type(s) of location(s) where the intervention occurred, including any necessary infrastructure or relevant features. |  | See study protocol. |
|  | **WHEN and HOW MUCH** |  |  |
| **8.** | Describe the number of times the intervention was delivered and over what period of time including the number of sessions, their schedule, and their duration, intensity or dose. | Page 09, Page 15 |  |
|  | **TAILORING** |  |  |
| **9.** | If the intervention was planned to be personalised, titrated or adapted, then describe what, why, when, and how. | Page 08-10 and Supplement: A4 | And see study protocol. |
|  | **MODIFICATIONS** |  |  |
| **10.^ǂ^** | If the intervention was modified during the course of the study, describe the changes (what, why, when, and how). | Planned modifications see Supplement, A4 |  |
|  | **HOW WELL** |  |  |
| **11.** | Planned: If intervention adherence or fidelity was assessed, describe how and by whom, and if any strategies were used to maintain or improve fidelity, describe them. | Page 15 |  |
| **12.^ǂ^** | Actual: If intervention adherence or fidelity was assessed, describe the extent to which the intervention was delivered as planned. | Page 15 |  |

** **Authors** - use N/A if an item is not applicable for the intervention being described. **Reviewers** – use ‘?’ if information about the element is not reported/not sufficiently reported.

† If the information is not provided in the primary paper, give details of where this information is available. This may include locations such as a published protocol or other published papers (provide citation details) or a website (provide the URL).

ǂ If completing the TIDieR checklist for a protocol, these items are not relevant to the protocol and cannot be described until the study is complete.

* We strongly recommend using this checklist in conjunction with the TIDieR guide (see *BMJ* 2014;348:g1687) which contains an explanation and elaboration for each item.

* The focus of TIDieR is on reporting details of the intervention elements (and where relevant, comparison elements) of a study. Other elements and methodological features of studies are covered by other reporting statements and checklists and have not been duplicated as part of the TIDieR checklist. When a **randomised trial** is being reported, the TIDieR checklist should be used in conjunction with the CONSORT statement (see [www.consort-statement.org](http://www.consort-statement.org)) as an extension of **Item 5 of the CONSORT 2010 Statement.** When a **clinical trial** **protocol** is being reported, the TIDieR checklist should be used in conjunction with the SPIRIT statement as an extension of **Item 11 of the SPIRIT 2013 Statement** (see [www.spirit-statement.org](http://www.spirit-statement.org)). For alternate study designs, TIDieR can be used in conjunction with the appropriate checklist for that study design (see [www.equator-network.org](http://www.equator-network.org)).

# A.20 Supplemental references

[1] Roos EM, Lohmander LS. The Knee injury and Osteoarthritis Outcome Score (KOOS): from joint injury to osteoarthritis. Health Qual Life Outcomes 2003; 1: 64. doi:10.1186/1477-7525-1-64

[2] Faul F, Erdfelder E, Lang A-G, et al. G*Power 3: a flexible statistical power analysis program for the social, behavioral, and biomedical sciences. Behav Res Methods 2007; 39: 175–191. doi:10.3758/BF03193146

[3] Abel R, Niederer D, Offerhaus C, et al. Effects of exercise prehabilitation before anterior cruciate ligament reconstruction on functional outcomes during pre- and postoperative rehabilitation - protocol for a single-blinded randomised controlled trial. Trials 2023; 24: 752. doi:10.1186/s13063-023-07776-1

[4] Roos EM, Roos HP, Lohmander LS, et al. Knee Injury and Osteoarthritis Outcome Score (KOOS)--development of a self-administered outcome measure. J Orthop Sports Phys Ther 1998; 28: 88–96. doi:10.2519/jospt.1998.28.2.88

[5] Frobell RB, Roos EM, Roos HP, et al. A randomized trial of treatment for acute anterior cruciate ligament tears. N Engl J Med 2010; 363: 331–342. doi:10.1056/NEJMoa0907797

[6] Beaton DE, Bombardier C, Guillemin F, et al. Guidelines for the process of cross-cultural adaptation of self-report measures. Spine (Phila Pa 1976) 2000; 25: 3186–3191. doi:10.1097/00007632-200012150-00014

[7] Salavati M, Akhbari B, Mohammadi F, et al. Knee injury and Osteoarthritis Outcome Score (KOOS); reliability and validity in competitive athletes after anterior cruciate ligament reconstruction. Osteoarthritis Cartilage 2011; 19: 406–410. doi:10.1016/j.joca.2011.01.010

[8] Frobell RB, Svensson E, Göthrick M, et al. Self-reported activity level and knee function in amateur football players: the influence of age, gender, history of knee injury and level of competition. Knee Surg Sports Traumatol Arthrosc 2008; 16: 713–719. doi:10.1007/s00167-008-0509-y

[9] Paradowski PT, Bergman S, Sundén-Lundius A, et al. Knee complaints vary with age and gender in the adult population. Population-based reference data for the Knee injury and Osteoarthritis Outcome Score (KOOS). BMC Musculoskelet Disord 2006; 7: 38. doi:10.1186/1471-2474-7-38

[10] Östenberg A, Roos EW, EKDAHL C, et al. Physical Capacity in Female Soccer Players - Does Age Make a Difference? Advances in Physiotherapy 2000; 2: 39–48. doi:10.1080/140381900443427

[11] Ageberg E, Forssblad M, Herbertsson P, et al. Sex differences in patient-reported outcomes after anterior cruciate ligament reconstruction: data from the Swedish knee ligament register. Am J Sports Med 2010; 38: 1334–1342. doi:10.1177/0363546510361218

[12] Müller S. Rückkehr zum Sport nach vorderer Kreuzbandruptur – Beurteilung durch Muskelfunktionstests. Sportverletz Sportschaden 2015; 29: 195. doi:10.1055/s-0035-1570422

[13] Filipa A, Byrnes R, Paterno MV, et al. Neuromuscular training improves performance on the star excursion balance test in young female athletes. J Orthop Sports Phys Ther 2010; 40: 551–558. doi:10.2519/jospt.2010.3325

[14] Plisky PJ, Rauh MJ, Kaminski TW, et al. Star Excursion Balance Test as a predictor of lower extremity injury in high school basketball players. J Orthop Sports Phys Ther 2006; 36: 911–919. doi:10.2519/jospt.2006.2244

[15] Thomeé R, Kaplan Y, Kvist J, et al. Muscle strength and hop performance criteria prior to return to sports after ACL reconstruction. Knee Surg Sports Traumatol Arthrosc 2011; 19: 1798–1805. doi:10.1007/s00167-011-1669-8

[16] Wilke C, Grimm L, Hoffmann B, et al. Funktionelle Tests als Entscheidungskriterium für die Rückkehr von Spielsportlern nach einer Ruptur des vorderen Kreuzbandes. Sportverletz Sportschaden 2018; 32: 171–186. doi:10.1055/a-0584-5280

[17] Webster KE, Feller JA, Lambros C. Development and preliminary validation of a scale to measure the psychological impact of returning to sport following anterior cruciate ligament reconstruction surgery. Phys Ther Sport 2008; 9: 9–15. doi:10.1016/j.ptsp.2007.09.003

[18] Tegner Y, Lysholm J. Rating systems in the evaluation of knee ligament injuries. Clin Orthop Relat Res 1985: 43–49

[19] Wirth B, Liffert F, Bruin ED de. Entwicklung und Evaluation einer deutschen Version des Lysholm-Scores zur Erfassung der Funktion nach einer Verletzung des vorderen Kreuzbands. Sportverletz Sportschaden 2011; 25: 37–43. doi:10.1055/s-0029-1245825

[20] Wirth B, Meier N, Koch PP, et al. Entwicklung und Evaluation einer deutschen Version der Tegner Aktivitätsskala zur Erfassung der Funktion nach einer Verletzung des vorderen Kreuzbands. Sportverletz Sportschaden 2013; 27: 21–27. doi:10.1055/s-0032-1330752
